# Enabling the controlled assembly of antibody conjugates with a loading of two modules without antibody engineering†
†Electronic supplementary information (ESI) available: ^1^H and ^13^C NMR spectra for all small molecules, ELISA, SDS-PAGE gels and UV-vis analysis (where applicable) for all bioconjugates. See DOI: 10.1039/c6sc03655d



**DOI:** 10.1039/c6sc03655d

**Published:** 2016-11-28

**Authors:** Maximillian T. W. Lee, Antoine Maruani, Daniel A. Richards, James R. Baker, Stephen Caddick, Vijay Chudasama

**Affiliations:** a Department of Chemistry , University College London , 20 Gordon Street , London , WC1H 0AJ , UK . Email: v.chudasama@ucl.ac.uk ; Tel: +44 (0)207 679 2077

## Abstract

The generation of antibody conjugates with a loading of two modules is desirable for a host of reasons. Whilst certain antibody engineering approaches have been useful in the preparation of such constructs, a reliable method based on a native antibody scaffold without the use of enzymes or harsh oxidative conditions has hitherto not been achieved. The use of native antibodies has several advantages in terms of cost, practicality, accessibility, time and overall efficiency. Herein we present a novel, reliable method of furnishing antibody conjugates with a loading of two modules starting from a native antibody scaffold.

## Abstract 2

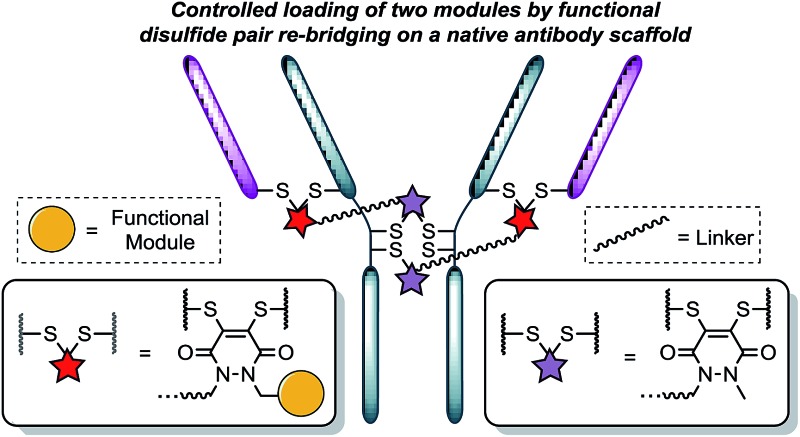
A novel reagent/strategy enables the controlled assembly of antibody conjugates with a loading of two modules without antibody engineering.

Antibody conjugates play an important role in a variety of applications, particularly in the field of diagnostics and therapeutics.^1,2^ In recent years, there has been significant interest in the area of antibody-drug conjugates (ADCs).^2^ ADCs comprise antibodies covalently attached to highly potent drugs using a linker conjugation technology.^2^ As therapeutics, they conceptually combine the specificity of antibodies, *i.e.* enabling discrimination between healthy and diseased tissue, with the cell-killing ability of cytotoxic drugs. This powerful and exciting class of targeted therapy has shown considerable promise in the treatment of various cancers with two US Food and Drug Administration approved ADCs currently on the market (Adcetris™ and Kadcyla™)^3,4^ and approximately 40 currently undergoing clinical evaluation.^5^ However, most of these ADCs exist as heterogeneous mixtures, which can result in a narrow therapeutic window and have major pharmacokinetic implications.^2,6^ In order for ADCs to achieve their full potential, sophisticated site-specific conjugation technologies to connect the drug to the antibody are increasingly being developed.^2^

Whilst a large number of reagents and strategies have been developed to create these next generation ADCs, novel strategies for site-specific conjugation continue to attract considerable interest.^2^ This is especially important as it is coming to light that specific requirements are essential for each particular ADC to operate at its optimum.^2^ Whilst engineered antibodies have worked well in meeting the demand for these tailor-made antibody conjugates, *e.g.* by using engineered cysteine residues, unnatural amino acids, selenocysteine or enzymatic conjugation,^7^ there is a requirement for methods that are based on native antibody modification. This is to ensure that technologies to make these “designer” ADCs are more accessible and cost-effective; engineered approaches often require significant optimisation on each antibody scaffold they are applied, as well not being accessible to a broad range of scientists.

It has recently come to light that in various tailor-made ADCs one of the most desirable ratios of drugs to antibody is two. The reason for this is that for certain hydrophobic drugs, *e.g.* pyrrolobenzodiazepines (PBDs), a loading of two is preferable as it provides a good balance between efficacy and pharmacokinetic profile (higher payload loading tends to result in too rapid clearance and lower loadings reduce efficacy). This argument is supported by the PBD-based ADCs that are currently in clinical trials.^2,8^ Whilst this particular challenge has to some extent been addressed by antibody engineering approaches, *e.g.* THIOMAbs for homogeneous DAR 2 conjugates, they are not readily accessible and there are issues associated with the methodology (*e.g.* potential for disulfide scrambling and the inefficiency of having to reduce, carefully re-oxidise and then conjugate).^2^ Thus, there is a need for a reliable method of constructing antibody conjugates with a loading of two entities starting from a native antibody construct. This is especially in the context of: (i) the rapid progression in the development of further hydrophobic drugs being used and developed in the field;^8a,b,9^ and (ii) the major attempts on native antibodies (*i.e.* based on the selective reduction of the Fab or hinge disulfides of an IgG1)^10g,11^ proving to lack broad applicability, as evidenced by the lack of uptake in the field, or having to employ harsh oxidation conditions or enzymes under specific conditions.^12^ Herein we detail the realisation of a reliable and reproducible strategy to make antibody conjugates with a loading of two starting from a native scaffold. It has significant advantages in terms of cost, practicality, accessibility, time and overall efficiency when compared to existing methods.

Recently, we have shown dibromopyridazinediones (diBrPDs) and dibromo/dithio-maleimides to be excellent candidates for the functional re-bridging of inter-chain disulfides in antibodies (Fig. 1). Moreover, the resulting bisthioether conjugates have been shown to be stable in blood plasma-mimicking conditions and retain activity plus selectivity *in vitro*.^10^ The dibromomaleimide platform has also been shown to be effective *in vivo* by Jackson and co-workers.^13^ Whilst this approach, as well as others,^10,13,14^ offer advances in terms of providing homogeneous DAR 4 conjugates starting from a non-engineered antibody scaffold, there has as yet been no translation of the technologies to form controlled DAR 2 conjugates. Nonetheless, we set out to explore if we could exploit the efficient functional re-bridging of disulfides with diBrPDs as a conduit to realise the goal of controlled DAR 2 conjugate formation starting from a native antibody scaffold. We envisaged that conjugation of two bis-dibromopyridazinediones (containing a single functional modality) with an appropriate linker length could “tie up” two pairs of the 4 disulfides on an IgG1 to allow the formation of a conjugate with an overall loading of two functional modules (see Fig. 2).

**Fig. 1:**
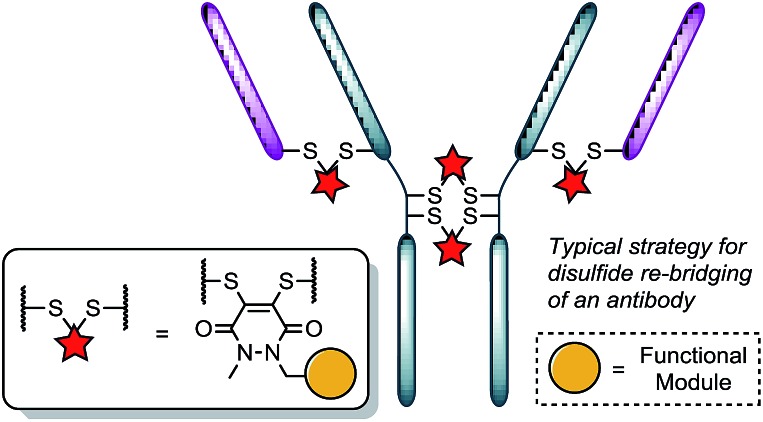
Illustration detailing the typical approach to functional disulfide re-bridging conjugates.

**Fig. 2:**
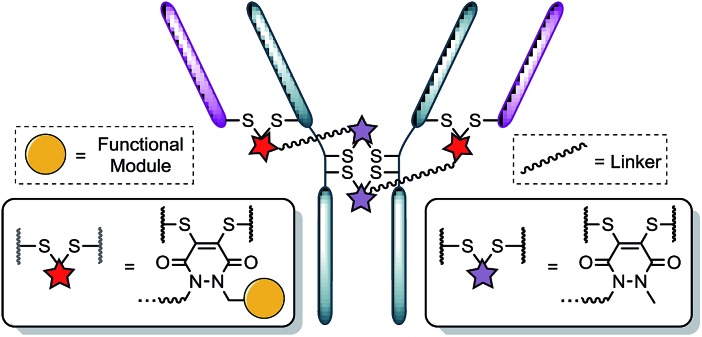
Illustration detailing a novel approach to achieving antibody conjugate with a loading of two. Each pair of red and purple stars per linker molecule are independently interchangeable, and other disulfide pairs (*e.g.* Fab–Fab, hinge–hinge) may be functionally re-bridged.

Our study began with the synthesis of an appropriate spaced bis-dibromopyridazinedione, bis-diBrPD **1**. As we were aiming to react a pair of disulfides using bis-diBrPD **1** it was rationalised from the outset that the linker length would be key. If too short, the bis-PD could not react with a pair of disulfides as it could not span the appropriate length; too long and it would increase the likelihood of undesirable intermolecular reaction(s) by losing the high local concentration effect – thus resulting in inefficient bridging of bis-diBrPD **1** across a pair of disulfides. In an effort to avoid these issues we designed a linker based on the spacing of the disulfides on an IgG1 for which a crystal structure has been obtained (∼16.7 Å = maximum linear distance).^14^^,^ ‡
‡Linear distance between disulfides determined by use of PyMol software. To ensure flexibility, and improve water solubility of the bis-dibromopyridazinedione, a PEG spacer was used to link together the PD bioconjugation sites. We installed a single alkyne on the bis-PD scaffold so as to eventually allow a loading of two modules, through the use of click chemistry, post-conjugation of bis-diBrPD **1** on a full antibody. The click handle was also positioned by design to be close to a PD-disulfide bridging site to minimise exposure of the clicked entity.

Compound **1** was synthesised by the route described in Scheme 1; in view of the maximum linear distance between inter-chain disulfides being ∼16.7 Å in the crystal structure of the IgG1, ∼25 Å was anticipated to be a suitable separation between the bridging sites when taking into account the resolution of the measurement,^15^ the flexibility of this region of the antibody and the PEG chain not being structurally linear. Initially, protected hydrazine **2** was formed *via* alkylation of diboc-hydrazine (see ESI for further details†). This species was then deprotected using TFA, and reacted with dibromomaleic anhydride under reflux in AcOH to afford methyl-PD **3**. Amide coupling of this PD with a mono-boc protected bis-amine resulted in the formation of PD linker reagent **4**. Finally, this species was deprotected and reacted with alkyne-PD **6** (prepared in an analogous manner to methyl-PD **3**) to afford target compound bis-diBrPD **1**. Whilst the overall yield for the synthesis was low (<5%) we do highlight that the chemistry is straightforward to carry out and that none of the steps were optimised as we were more interested in exploring the novel chemical biology methodology.

**Scheme 1:**
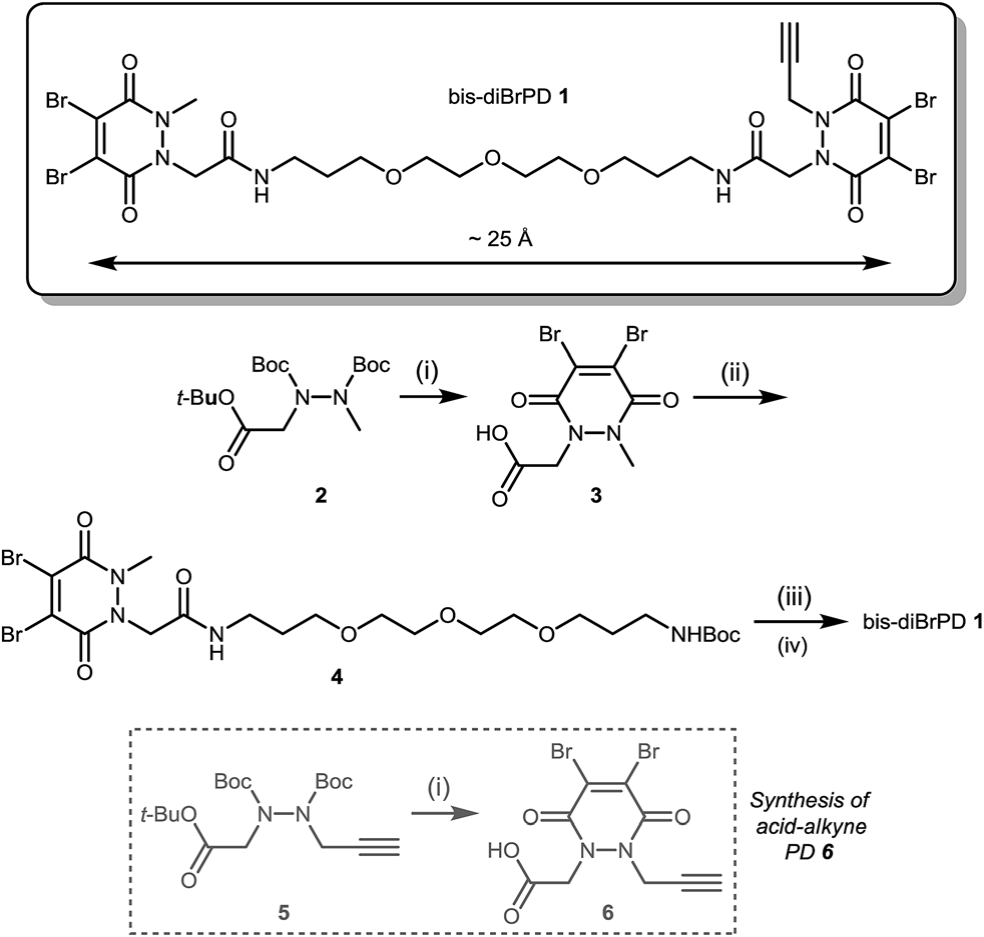
Synthesis of bis-diBrPD **1**. Reagents and conditions: (i) TFA, CH_2_Cl_2_, 21 °C, 1 h; dibromomaleic anhydride, AcOH, reflux, 2 h; (ii) *tert*-butyl (3-(2-(2-(3-aminopropoxy)ethoxy)ethoxy)propyl)carbamate, CDI, DMF, 21 °C, 12 h; (iii) TFA/DCM, 21 °C, 30 min; (iv) HATU, DIPEA, DMF, acid-alkyne PD **6** 21 °C, 16 h.

Following the successful synthesis, the conjugation of **1** on a full antibody was appraised on Herceptin™, a clinically improved immunoglobulin used for the treatment of breast cancer and the antibody component of FDA-approved ADC Kadcyla™.^4^ On account of pyridazinediones having a significant extinction coefficient at a distinct wavelength to other entities in the corresponding antibody conjugate, UV-vis was considered to be an appropriate method to analyse PD loading on the antibody. To our delight, after minimal optimisation, reaction of Herceptin™ with bis-diBrPD **1** gave a pyridazinedione-to-antibody ratio (PDAR) of 4.0 with complete functional re-bridging confirmed by SDS-PAGE (including under TCEP reducing conditions). This result supported our hypothesis, as it suggested each bis-diBrPD had re-bridged two disulfides; *i.e.* only such a result could have yielded the observed SDS-PAGE profile and PDAR. Despite the challenges of using mass spec for characterisation of antibody conjugates,^16^ especially for disulfide modified conjugates,^13^ we obtained mass spec data for conjugate **7** (see ESI for details†), which further verified our observation; *i.e.* we observed two additions of bis-diBrPD **1** to Herceptin™ (see representation in Fig. 3).

**Fig. 3:**
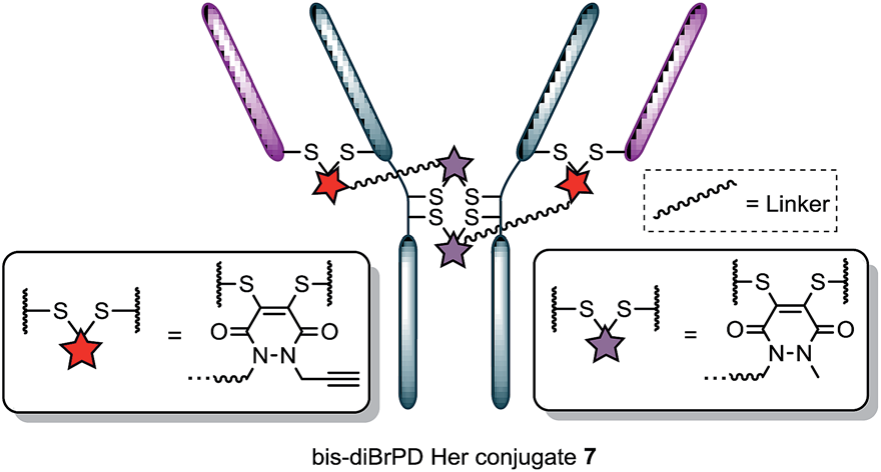
Illustration of bis-diBrPD her conjugate **7**. Each pair of red and purple stars per linker molecule are independently interchangeable, and other disulfide pairs (*e.g.* Fab–Fab, hinge–hinge) may be functionally re-bridged.

With conjugate **7** in hand, we next appraised what the loading would be if we were to click on a fluorophore-azide. Owing to its favourable optical properties and our previous experience with the azide,^10*i*^ we chose to click on Alexa Fluor^®^ 488 azide. To ensure that all available pendant alkynes on conjugate **7** would be reacted, an excess of Alexa Fluor^®^ 488 azide was used in the click reaction (20 eq.). Gratifyingly, these conditions resulted in the formation of a conjugate where the loading of the Alexa Fluor^®^ 488 dye on the antibody conjugate was 2.0. Furthermore, the result was highly reproducible with the click reaction not affecting PDAR or promoting antibody degradation by SDS-PAGE. In addition to this, pre-click modification (*i.e.* carrying out the click reaction prior to bioconjugation) afforded similar results (see ESI for details†).

It is also noteworthy that the final conjugate retained binding activity by ELISA (Fig. 4), even when compared to classical diBrPD conjugation (see ESI for details†).^10c,i^ More than this, the reactions were highly reproducible, with as many as seven attempts showing PDARs in the range of 3.9–4.1 and Alexa Fluor^®^ 488 loadings in the range of 1.9–2.1. Finally, an ADC with a DAR of 2.0, based on the “click” conjugation of an azide functionalised doxorubicin^10*i*^ to conjugate **7**, was prepared in a facile manner owing to the modular nature of the chemistry. These results thus provide the first examples of forming a conjugate with a controlled loading of 2.0 on a native antibody scaffold in a facile and reliable manner.

**Fig. 4:**
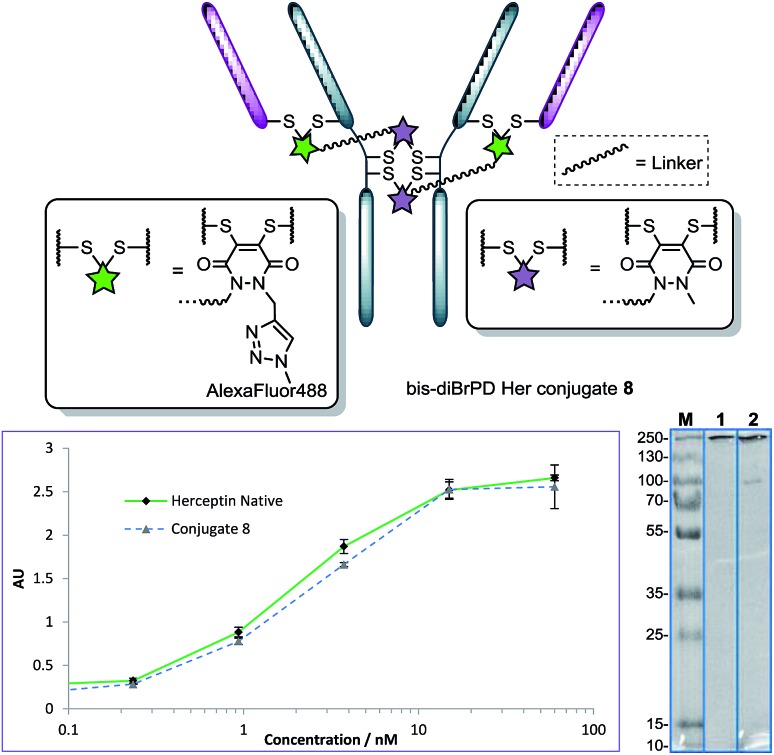
Structure of conjugate **8** (each pair of green and purple stars per linker molecule are independently interchangeable, and other disulfide pairs (*e.g.* Fab–Fab, hinge–hinge) may be functionally re-bridged); ELISA and SDS-PAGE gel (M: molecular weight marker; **1**: native Herceptin™; **2**: conjugate **8**) of conjugate **8** with native Herceptin™ control.

Not content with providing conjugates with a controlled loading of two modules, we next turned our attention to the synthesis of an antibody conjugate with a loading of four to showcase the flexibility of our strategy. This would also serve as further proof of our disulfide pair functional re-bridging hypothesis as well as provide a novel conjugation strategy for making antibody conjugates with a loading of four. To this end, we synthesised bis-diBrPD **9** in a similar manner to which bis-diBrPD **1** was formed (see Scheme 2).

**Scheme 2:**
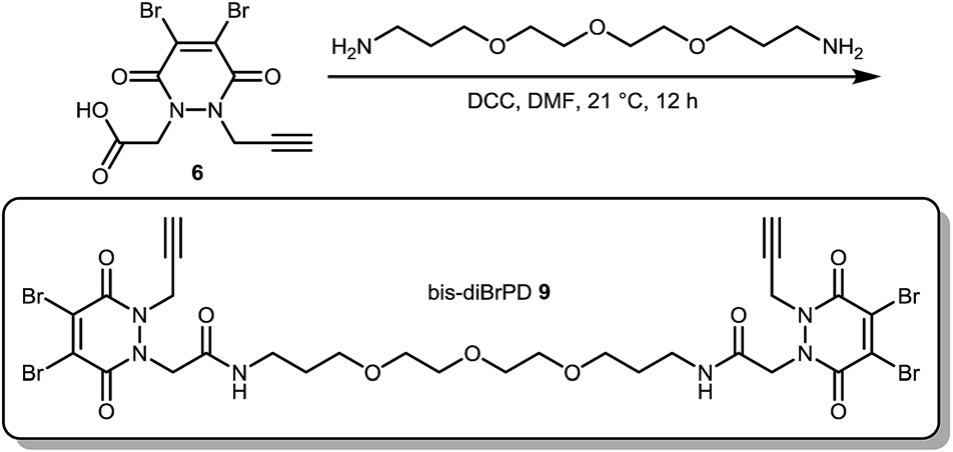
Synthesis of bis-diBrPD **9**.

Pleasingly, application of the optimised conditions for functional re-bridging, in the formation conjugate **7**, to reagent **9** on Herceptin™ afforded a conjugate with a PDAR 3.9 with all disulfides of the antibody functionalised (Fig. 5, see ESI for further details†). Moreover, reaction with an excess of Alexa Fluor® 488 azide resulted in a fluorophore loading of 3.9 (Fig. 5, see ESI for details†). This, in combination with the SDS-PAGE data, further verifies the bridging mode of these first-in-class reagents, and ELISA data again showed binding to be unaffected by the strategy. Moreover, it showcases how this novel class of reagent can act as a branch point for the construction of antibody conjugates with distinct and controlled module loadings of two and four.

**Fig. 5:**
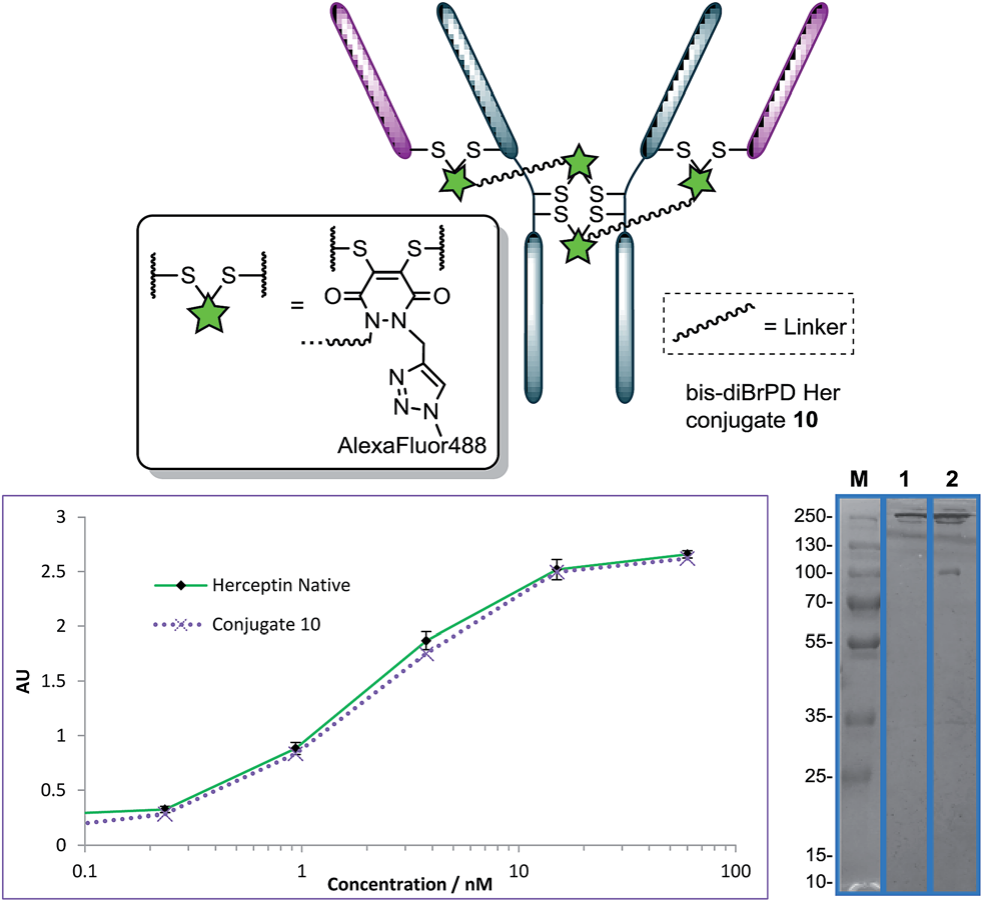
Structure of conjugate **10** (other disulfide pairs (*e.g.* Fab–Fab, hinge–hinge) may be functionally re-bridged); ELISA and SDS-PAGE gel (M: molecular weight marker; **1**: native Herceptin™; **2**: conjugate **10**) of conjugate **10** with native Herceptin™ control.

## Conclusions

In conclusion, we have provided a first-in-class strategy for enabling the controlled, reproducible loading of two modules onto a native antibody scaffold. This was enabled by the successful synthesis and application of an entity, containing one “click” handle, which functionally re-bridges two pairs of disulfide bonds on an IgG1. Subsequent reaction of the formed conjugate with an azide-fluorophore and azide-drug showed that the controlled loading of two modules was feasible and reproducible. The strategy, starting from the same branch point, was also readily adapted for the formation of a conjugate with a loading of four modules. Furthermore, all final conjugates were shown to retain binding by ELISA and antibody-PD conjugates have previously been shown to be biologically functional and stable.^10i–k^ We believe this simple yet elegant approach to facilitate the loading of two modules starting from a native antibody scaffold will find application in multiple fields where the controlled loading of lower than four entities is desirable (*e.g.* antibody conjugates bearing highly hydrophobic modules) and especially in laboratories where antibody engineering techniques are not accessible, too expensive or not practical.
