# Supplementary material for: Enabling the controlled assembly of antibody conjugates with a loading of two modules without antibody engineering
Source: Chem Sci. 2016 Nov 28;8(3):2056–60. doi: 10.1039/c6sc03655d (PMC5399535; doi:10.1039/c6sc03655d)
Supplement: Supplementary file 1 [file SC-008-C6SC03655D-s001.pdf]

Electronic Supplementary Material (ESI) for Chemical Science.
This journal is © The Royal Society of Chemistry 2016

Enabling the controlled assembly of antibody conjugates with a loading of two modules without antibody engineering

Maximillian T. W. Lee, Antoine Maruani, Daniel A. Richards, James R. Baker, Stephen Caddick and Vijay Chudasama*

Department of Chemistry, University College London, 20 Gordon Street, London, WC1H OAJ, UK

* *Tel: +44 (0)20 76792077; E-mail:* v.chudasama@ucl.ac.uk

General Experimental

All reagents were purchased from Aldrich, AlfaAesar, Sino Biologial Inc or Lumiprobe and were used as received. Where described below Pet. refers to petroleum ether (40–60 °C). All reactions were monitored by thin-layer chromatography (TLC) on pre-coated SIL G/UV254 silica gel plates (254 μm) purchased from VWR. Flash column chromatography was carried out with Kiesegel 60M 0.04/0.063 mm (200–400 mesh) silica gel. ^{1}H and ^{13}C NMR spectra were recorded at ambient temperature on a Bruker Avance 300 instrument operating at a frequency of 300 MHz for ^{1}H and 75 MHz for ^{13}C, a Bruker Avance 500 instrument operating at a frequency of 500 MHz for ^{1}H and 125 MHz for ^{13}C, and a Bruker Avance 600 instrument operating at a frequency of 600 MHz for ^{1}H and 150 MHz for ^{13}C in $CDCl_3$ or CD_3OD (as indicated below). The chemical shifts (δ) for ^{1}H and ^{13}C are quoted relative to residual signals of the solvent on the ppm scale. ^{1}H NMR peaks are reported as singlet (s), doublet (d), triplet (t), quartet (q), doublet of doublets (dd), triplet of doublets (td), doublet of triplets (dt), triplet of triplets (tt) m (multiplet) and br (broad). Coupling constants (*J* values) are reported in Hertz (Hz) and are H-H coupling constants unless otherwise stated. Signal multiplicities in ^{13}C NMR were determined using the distortionless enhancement by phase transfer (DEPT) spectral editing technique. Infrared spectra were obtained on a Perkin Elmer Spectrum 100 FTIR Spectrometer operating in ATR mode with frequencies given in reciprocal centimetres (cm^{-1}). Melting points were measured with a Gallenkamp apparatus and are uncorrected. All bioconjugation reactions were carried out in triplicate.

UV-Vis spectroscopy

UV-Vis spectroscopy was used to determine protein concentrations, pyridazinedione to antibody ratios (PDAR), fluorophore to antibody ratios (FAR) and drug to antibody ratios (DAR) using a nanodrop ND-1000 spectrophotometer and a Varian Cary 100 Bio UV-Visible spectrophotometer operating at 21 °C. Sample buffer was used as blank for baseline correction with extinction coefficients; ε_{280} = 225,000 M^{-1} cm^{-1} for Herceptin™, ε_{335} = 9,100 M^{-1} cm^{-1} for pyridazinedione scaffolds, ε_{493} = 75,000 M^{-1} cm^{-1} for Alexafluor 488™ (AF488) and ε_{495} = 8,030 M^{-1} cm^{-1} for doxorubicin. The correction factors for pyridazinedione scaffolds, AF488 and doxorubicin at 280 nm are 0.25, 0.11 and 0.72, respectively. Calculations are performed according to that previously described.[1]

SDS-PAGE gels

Non-reducing glycine-SDS-PAGE at 12% acrylamide gels were performed following standard lab procedures, unless stated otherwise. A 6% stacking gel was used and a broad-range MW marker (10–250 kDa, Prestained PagerulerPlus Protein Standards, Bio-Rad) was co-run to estimate protein weights. Samples (15 µL at ~12 µM construct) were mixed with loading buffer (3 µL, composition for 6 × SDS: 1 g SDS, 3 mL glycerol, 6 mL 0.5 M Tris buffer pH 6.8, 2 mg bromophenol blue in 10 mL) and heated at 75 °C for 3 min. The gels were run at 30 mA for 50 min in 1 × SDS running buffer. The gels were stained with Coomassie dye.

Protein LC-MS

Full Herceptin™ mAb conjugate **7** and Herceptin™ mAb were prepared for analysis by repeated diafiltration into ammonium acetate buffer (50 mM ammonium acetate, pH 6.9) using VivaSpin sample concentrators (GE Healthcare, 10,000 MWCO) to a concentration of 6.6 µM (1.0 mg·ml^{-1}). Following this, PNGase F (1 µL, 15,000 units·mL^{-1}, in 20 mM Tris-HCl, 50mM NaCl, 5 mM EDTA, pH 7.5) (purchased from New England BioLabs Inc.) was added and the resultant solution was incubated at 37 °C for 16 h. After this time the solution was diluted to 0.7 µM (1.0 mg·ml^{-1}) in water and submitted to the UCL Chemistry Mass Spectrometry Facility at the Chemistry Department, UCL for analysis of antibody conjugates on the Agilent 6510 QTOF LC-MS system (Agilent, UK). 10 µL of each sample was injected onto a PLRP-S, 1000A, 8 µM, 150 mm x 2.1 mm column, which was maintained at 60 °C. The separation was achieved using mobile phase A (5% MeCN in 0.1% formic acid) and B (95% MeCN, 5% water 0.1% formic acid) using a gradient elution. The column effluent was continuously electrosprayed into capillary ESI source of the Agilent 6510 QTOF mass spectrometer and ESI mass spectra were acquired in positive electrospray ionisation (ESI) mode using the m/z range 1,000−8,000 in profile mode. The raw data was converted to zero charge mass spectra using maximum entropy deconvolution algorithm over the region 12.3–16.8 min with MassHunter software (version B.07.00).

Tri-*tert*-butyl 2-methylhydrazine-1,1,2-tricarboxylate[1]

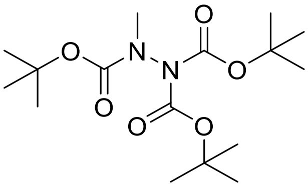

To a solution of methylhydrazine (1.00 g, 1.14 mL, 21.7 mmol), NEt_3 (4.34 g, 6.04 mL, 43.4 mmol) and DMAP (260 mg, 2.17 mmol) in CH_2Cl_2 (75 mL) was added Boc_2O (18.9 g, 86.6 mmol), and the reaction mixture stirred at 20 °C for 72 h. After this time, the reaction mixture was diluted with H_2O (80 mL), extracted with EtOAc (3 × 60 mL), and the combined organic layers dried ($MgSO_4$) and concentrated *in vacuo*. The crude residue was purified by flash column chromatography (20% EtOAc/petrol) to afford tri-*tert*-butyl 2-methylhydrazine-1,1,2-tricarboxylate (7.43 g, 21.5 mmol, 99%) as a yellowish oil: ^{1}H NMR (600 MHz, $CDCl_3$) (major rotamer) δ 3.05 (s, 3H), 1.51–1.43 (m, 27H); ^{13}C NMR (150 MHz, $CDCl_3$) (major rotamer) δ 154.0 (C), 150.1 (C) 83.4 (C), 81.4 (C), 35.7 (CH_3), 28.3 (CH_3), 28.1 (CH_3); LRMS (ES+) 369 (100, $[M+Na]^+$).

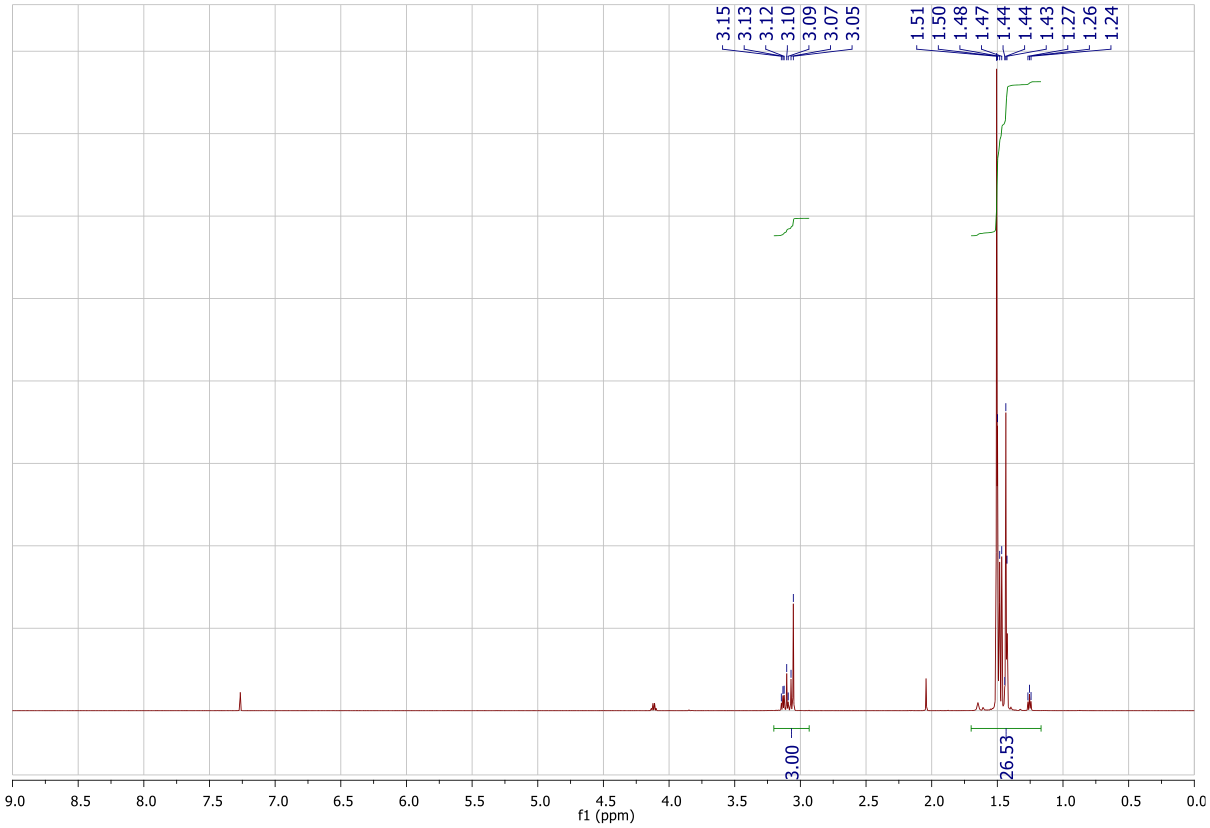

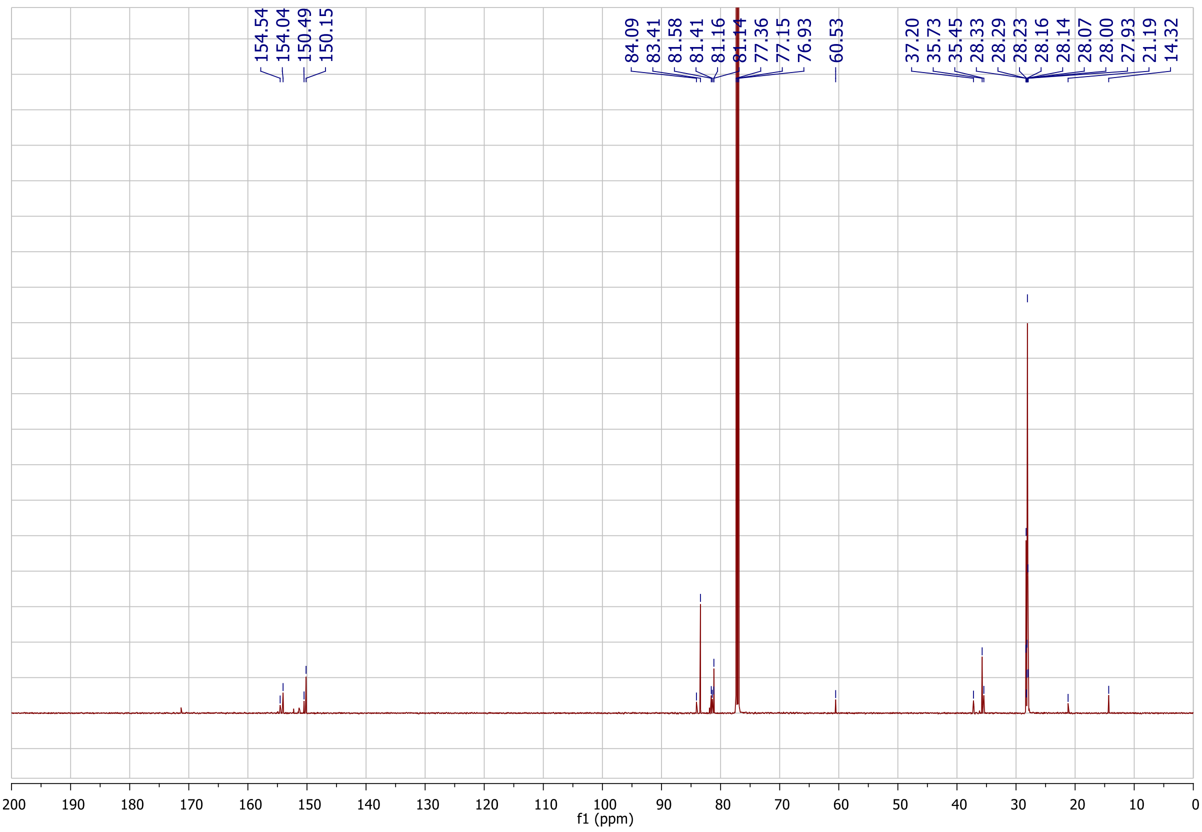

Figure **S1**. ^{1}H and ^{13}C NMR data for tri-*tert*-butyl 2-methylhydrazine-1,1,2-tricarboxylate.

Di-*tert*-butyl 1-methylhydrazine-1,2-dicarboxylate[1]

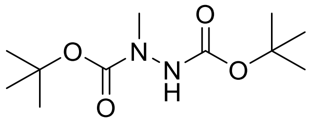

To a solution of tri-*tert*-butyl 2-methylhydrazine-1,1,2-tricarboxylate (2.0 g, 5.8 mmol) in dry MeCN (15 mL) was added $Mg(ClO_4)_2$ (0.27 g, 1.21 mmol), and the reaction mixture stirred at 20 °C for 1 h. After this time, the reaction mixture was diluted with 10% aq. citric acid (20 mL) and Et_2O (15 mL), extracted with Et_2O (3 × 20 mL), and the combined organic layers were dried ($MgSO_4$) and concentrated *in vacuo*. The crude residue was purified by flash column chromatography (15% EtOAc/petrol) to afford di-*tert*-butyl 1-methylhydrazine-1,2-dicarboxylate (1.3 g, 5.2 mmol, 89%) as a white solid: m.p. 54–55 °C; ^{1}H NMR (600 MHz, $CDCl_3$) (major rotamer) δ 6.40 (br. s, 1H), 3.11 (s, 3H), 1.48–1.45 (m, 18H); ^{13}C NMR (150 MHz, $CDCl_3$) (major rotamer) δ 155.9 (C), 155.3 (C) 81.3 (C), 81.1 (C), 37.6 (CH_3), 28.3 (CH_3), 28.1 (CH_3); IR (solid) 3316, 2978, 2932, 1701 cm^{-1}; LRMS (ES+) 269 (100, $[M+Na]^+$); HRMS (ES+) calcd. for $C_{11}H_{22}N_2O_4Na$ $[M+Na]^+$ 269.1477, observed 269.1476.

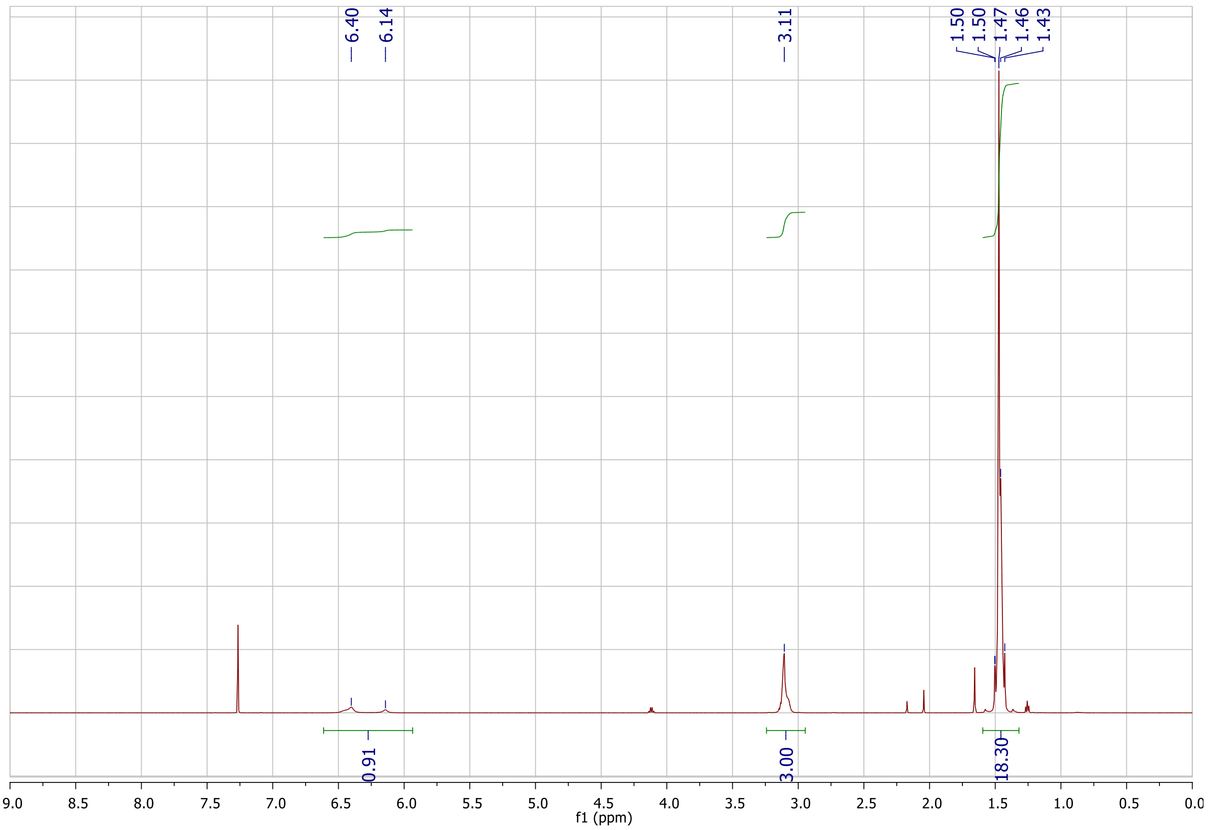

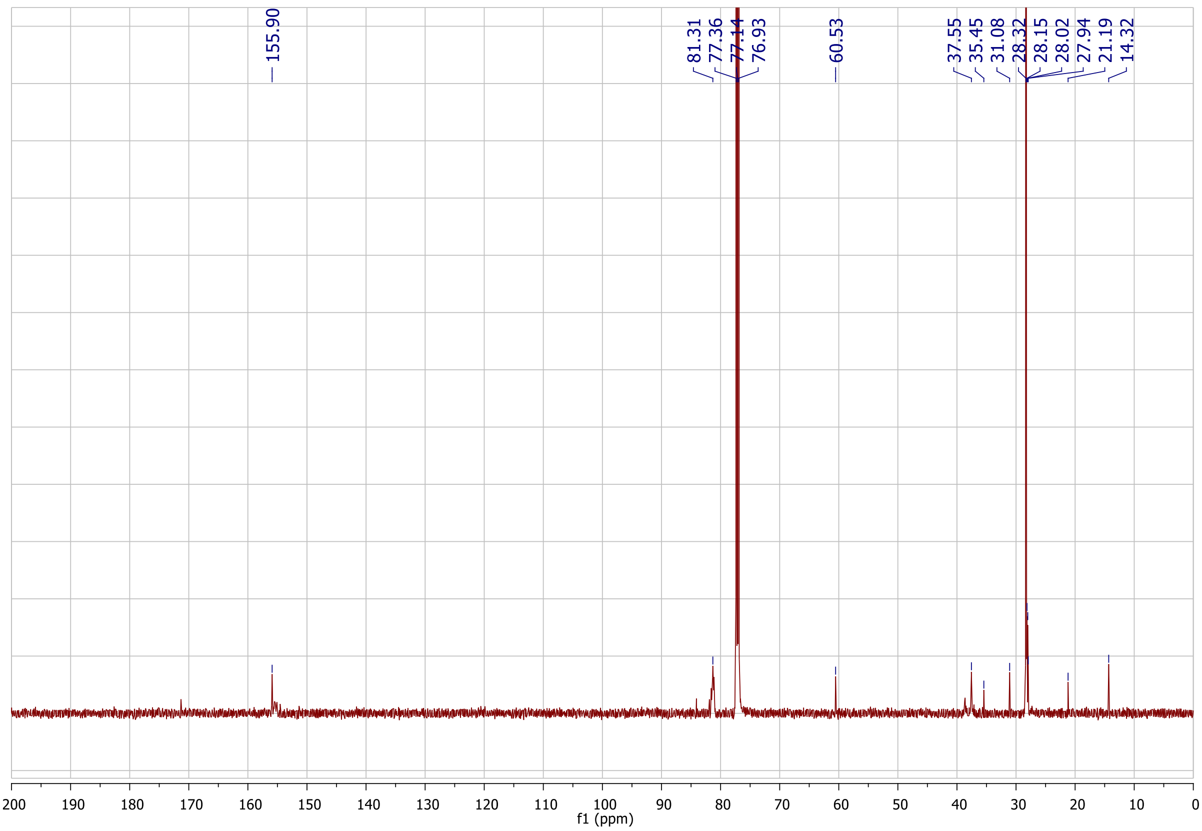

Figure **S2**. ^{1}H and ^{13}C NMR data for di-*tert*-butyl 1-methylhydrazine-1,2-dicarboxylate.

Di-*tert*-butyl 1-(2-(*tert*-butoxy)-2-oxoethyl)-2-methylhydrazine-1,2-dicarboxylate (2)[1]

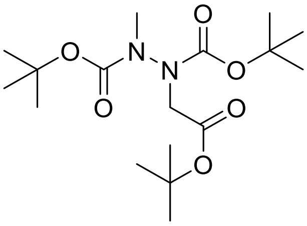

To a solution of di-*tert*-butyl 1-methylhydrazine-1,2-dicarboxylate (0.94 g, 3.8 mmol) in DMF (20 mL) was added caesium carbonate (1.86 g, 5.7 mmol) and then *tert*-butyl bromoacetate (1.1 g, 0.84 mL, 5.7 mmol), and the reaction mixture stirred at 20 °C for 16 h. After this time, the reaction mixture was diluted with H_2O (50 mL), extracted with Et_2O (4 × 50 mL), the combined organic layers washed with sat. aq. LiCl (2 × 30 mL), dried ($MgSO_4$), and concentrated *in vacuo*. Purification by flash column chromatography (10% Et_2O/petrol) yielded di-*tert*-butyl 1-(2-(*tert*-butoxy)-2-oxoethyl)- 2-methylhydrazine-1,2-dicarboxylate **2** (1.3 g, 3.7 mmol, 98%) as a colourless oil: ^1H NMR (600 MHz, $CDCl_3$) δ 4.73–4.04 (m, 2H), 3.68–3.10 (m, 3H), 1.54–1.39 (m, 27H); ^{13}C NMR (150 MHz, $CDCl_3$) (major rotamer) δ 169.2 (C), 155.2 (C), 81.9 (C), 81.6 (C), 81.1 (C), 52.7 (CH_2), 36.8 (CH_3), 28.4 (CH_3), 28.3 (CH_3), 28.2 (CH_3); IR (thin film) 2978, 1748 cm^{-1}; LRMS (ES+) 361 (100, $[M+H]^+$); HRMS (ES+) calcd for $C_{17}H_{33}O_6N_2$ $[M+H]^+$ 361.2339, observed 361.2333.

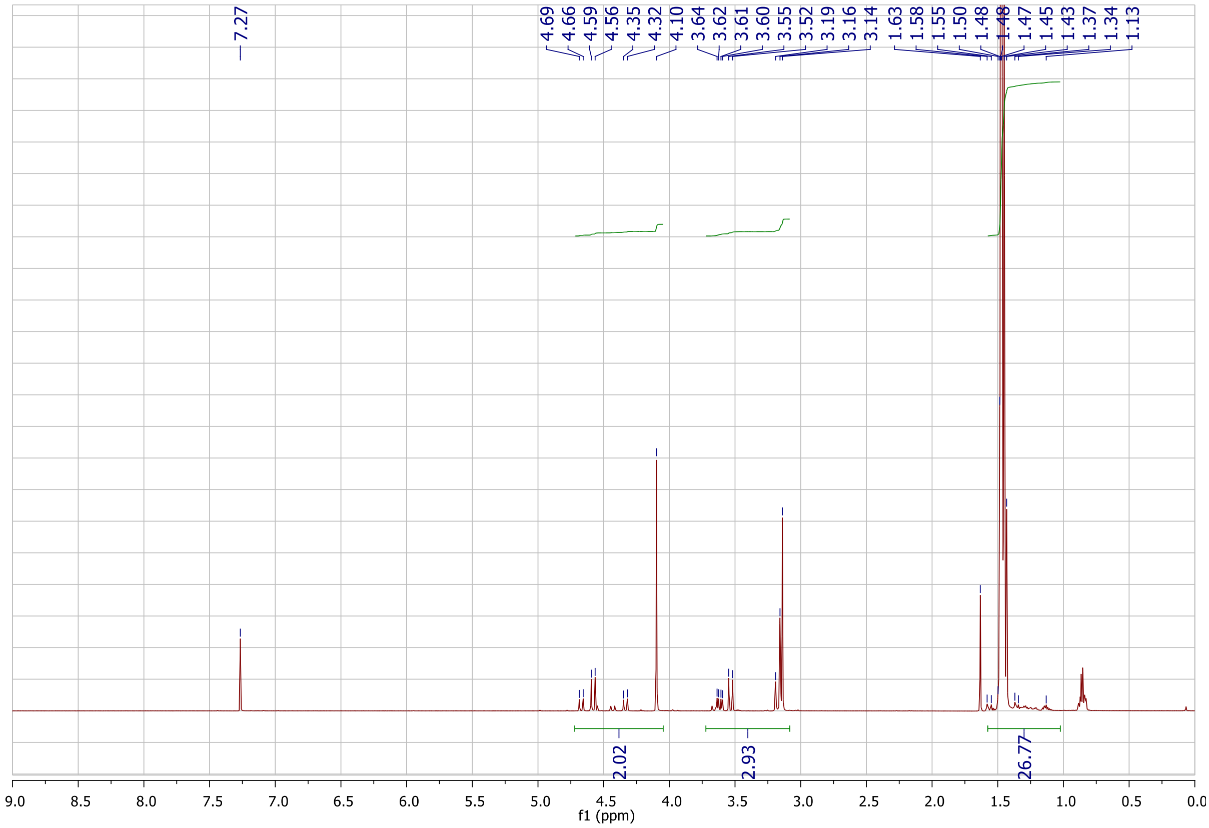

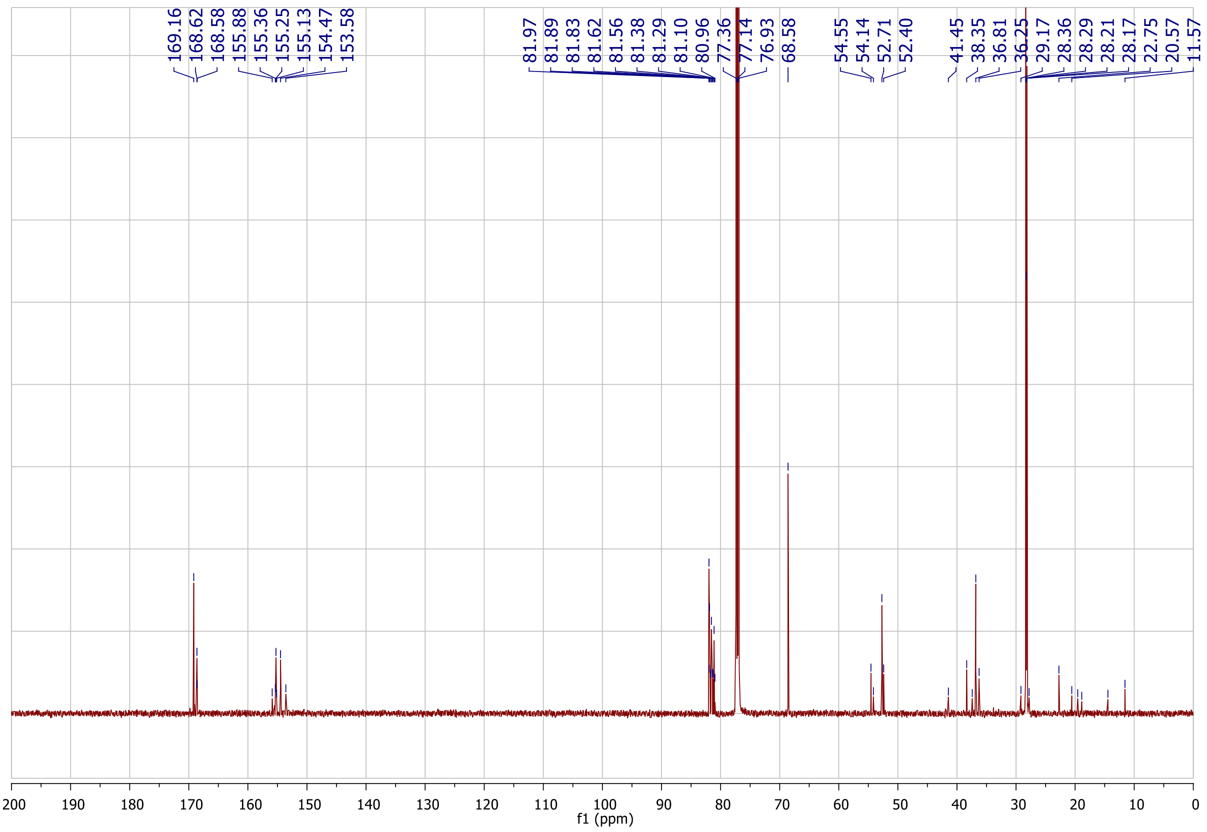

Figure **S3**. ^{1}H and ^{13}C NMR data for di-*tert*-butyl 1-(2-(*tert*-butoxy)-2-oxoethyl)-2-methylhydrazine-1,2-dicarboxylate **2**.

2-(4,5-Dibromo-2-methyl-3,6-dioxo-3,6-dihydropyridazin-1(2*H*)-yl)acetic acid (3)[1]

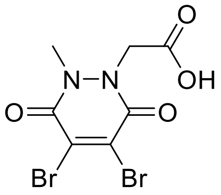

To a solution of di-*tert*-butyl 1-(2-(*tert*-butoxy)-2-oxoethyl)-2-methylhydrazine-1,2-dicarboxylate **2** (1.0 g, 2.8 mmol) in CH_2Cl_2 (10 mL) was added TFA (10 mL), and the reaction mixture stirred at 20 °C for 2 h. After this time, all volatile materials were removed *in vacuo*. The crude residue was added to a solution of 2,3-dibromomaleic anhydride (0.75 g, 2.8 mmol) in glacial AcOH (40 mL), and the reaction mixture stirred at 20 °C for 16 h before raising the temperature to 130 °C for 16 h. After this time, the reaction mixture was concentrated *in vacuo*, and purification of the crude residue by flash column chromatography (3% MeOH/CH_2Cl_2 with 1% AcOH) yielded 2-(4,5-dibromo-2-methyl-3,6-dioxo-3,6-dihydropyridazin-1(2*H*)-yl)acetic acid **3** (0.65 g, 1.9 mmol, 73%) as a white solid: m.p. 210–214 °C; ^{1}H NMR (600 MHz, MeOD) δ 4.96 (s, 2H), 3.62 (s, 3H); ^{13}C NMR (150 MHz, MeOD) δ 170.2 (C), 154.8 (C), 154.0 (C), 137.4 (C), 135.7 (C), 49.5 (CH_2), 35.0 (CH_3); IR (solid) 3023, 2969, 1731, 1662 cm^{-1}; LRMS (ES−) 341 (50, [M^{81}Br^{81}Br-H]$^-$), 339 (100, [M^{81}Br^{79}Br-H]$^-$), 337 (50, [M^{79}Br^{79}Br-H]$^-$); HRMS (ES−) calcd for $C_7H_5N_2O_4Br_2$ [M^{79}Br^{79}Br-H]$^-$ 336.8538, observed 336.8540.

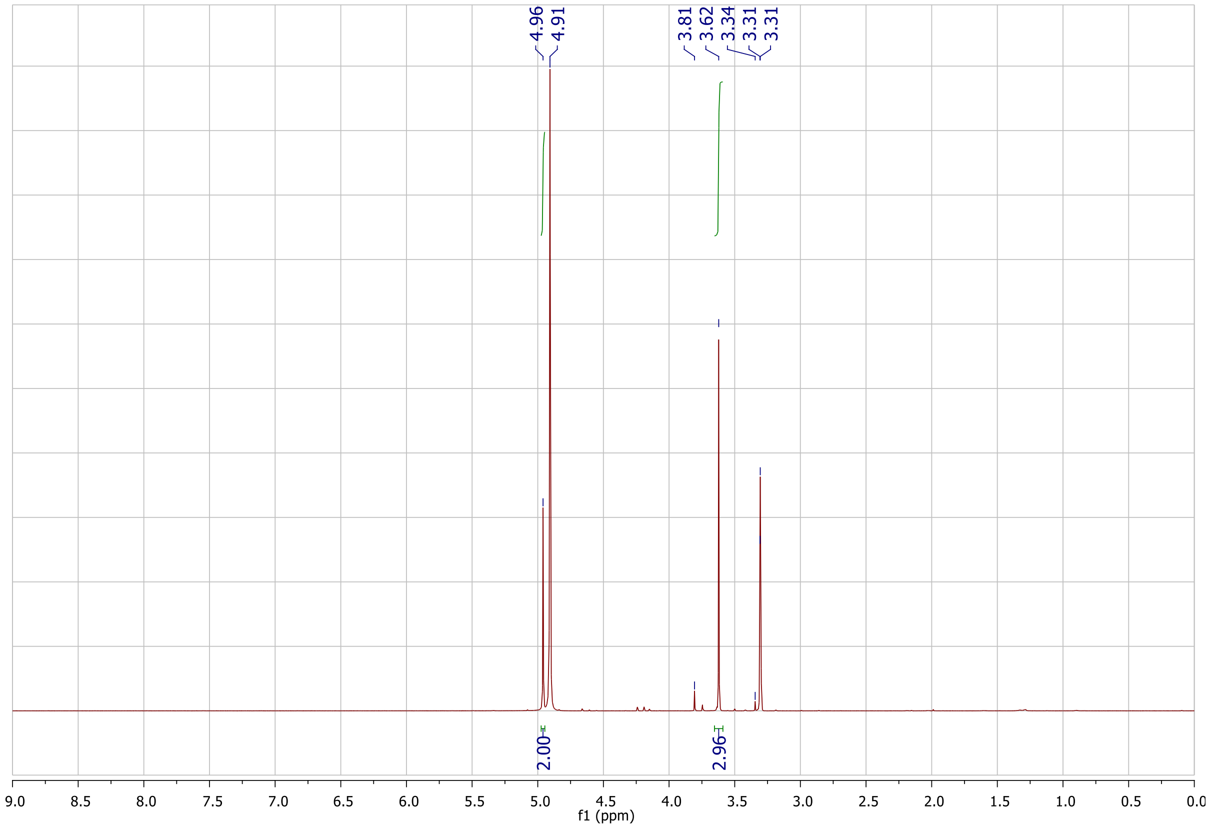

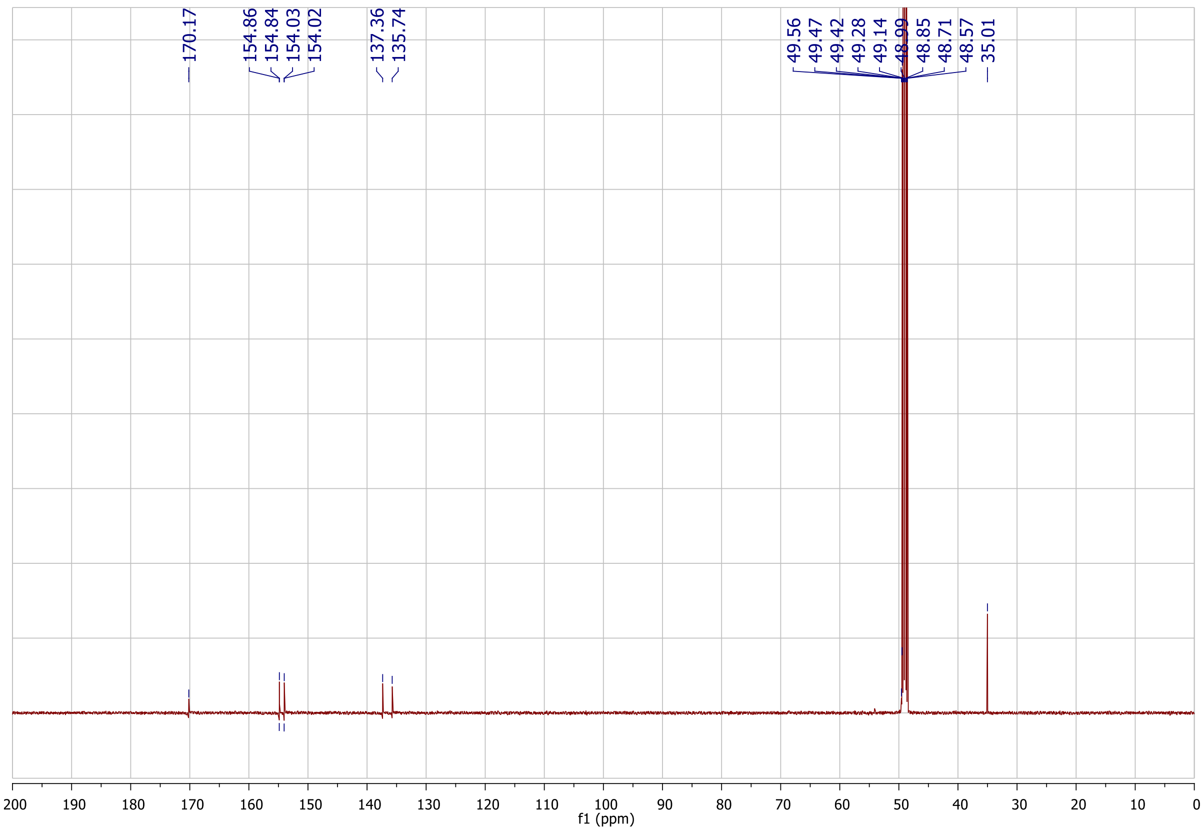

Figure **S4**. ^{1}H and ^{13}C NMR data for 2-(4,5-dibromo-2-methyl-3,6-dioxo-3,6-dihydropyridazin-1(2*H*)-yl)acetic acid **3**.

Di-*tert*-butyl-1-(prop-2-yn-1-yl)hydrazine-1,2-dicarboxylate[2]

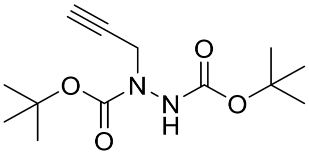

To a solution of di-*tert*-butyl hydrazine-1,2-dicarboxylate (2.00 g, 8.62 mmol) in a mixture of toluene (10 mL) and 5% aq. NaOH (10 mL) were added tetra-*n*-butylammonium bromide (87 mg, 0.26 mmol) and then propargyl bromide (3.08 g, 25.86 mmol). The reaction mixture was stirred at 20 °C for 16 h. After this time, H_2O (20 mL) was added and the mixture was extracted with EtOAc (3 × 15 mL). The combined organic layers were washed with brine (15 mL), dried ($MgSO_4$), and concentrated *in vacuo*. Purification of the crude residue by flash column chromatography (20% EtOAc/petrol) yielded di-*tert*-butyl 1-(prop-2-yn- 1-yl)hydrazine-1,2-dicarboxylate (1.93 g, 7.14 mmol, 83%) as a white solid: m.p. 101–103 °C; ^1H NMR (500 MHz, $CDCl_3$) δ 6.47 (br. s, 0.78H), 6.18 (br. s, 0.22H,), 4.27 (s, 2H), 2.24 (t, J = 2.4 Hz, 1H), 1.48 (s, 18H); ^{13}C NMR (150 MHz, $CDCl_3$) δ 155.0 (C), 82.2 (C), 81.7 (C), 79.0 (C), 77.7 (C), 72.5 (CH), 39.5 (CH_2), 28.5 (CH_3), 28.5 (CH_3); IR (solid) 3310, 2109, 1703 cm^{-1}.

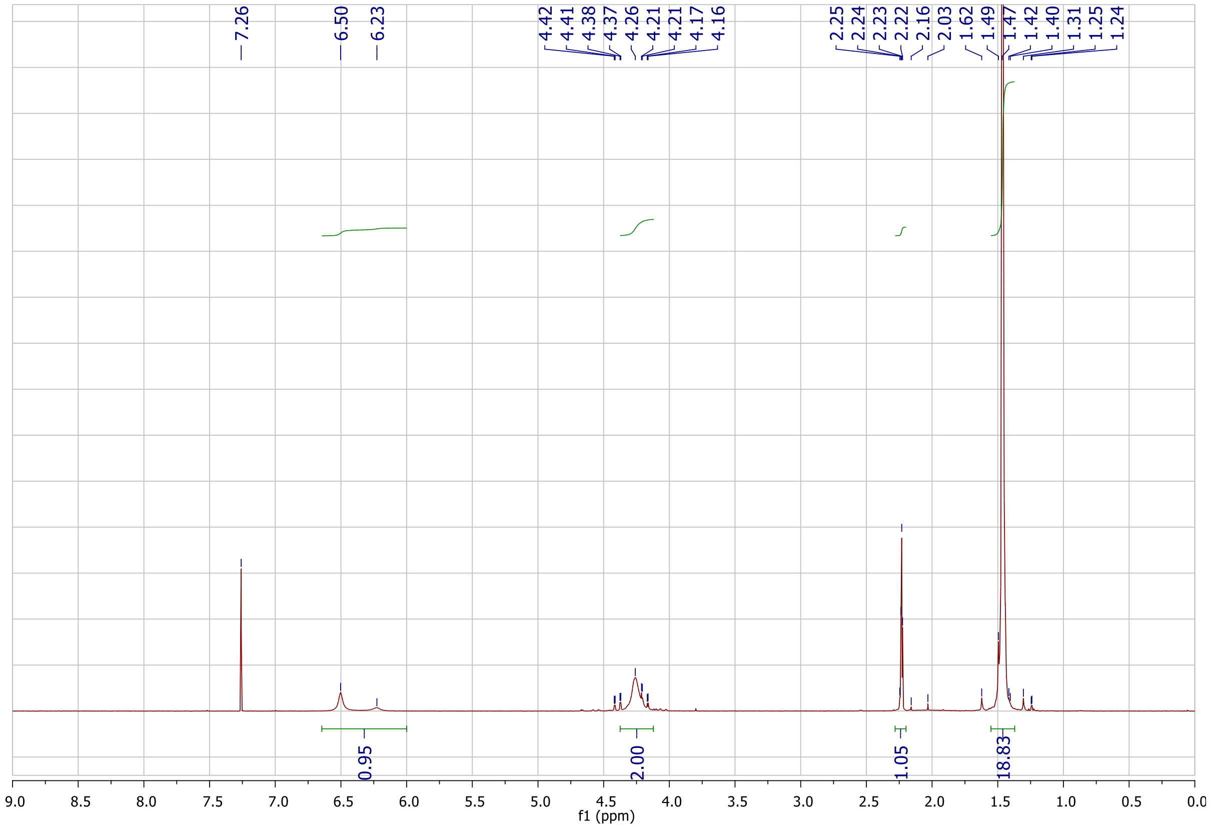

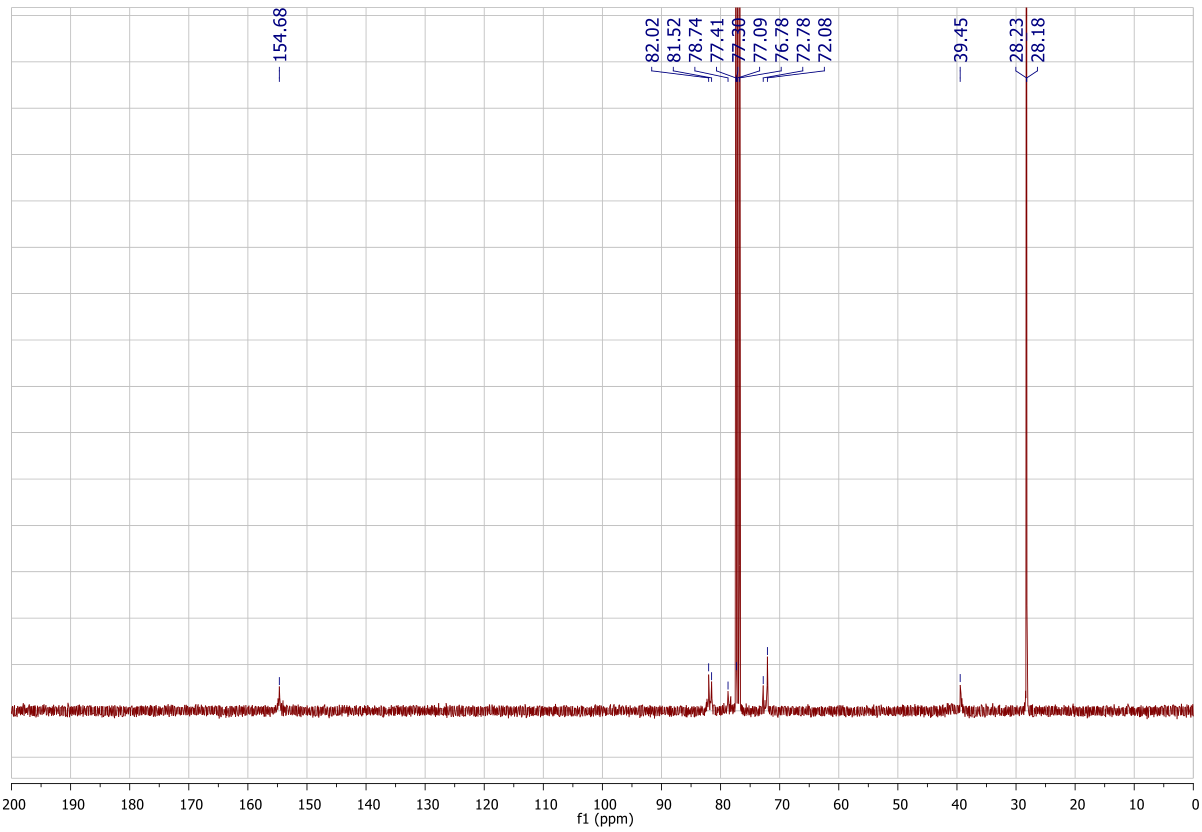

Figure **S5**. ^{1}H and ^{13}C NMR data for di-*tert*-butyl-1-(prop-2-yn-1-yl)hydrazine-1,2-dicarboxylate.

Di-*tert*-butyl 1-(2-(tert-butoxy)-2-oxoethyl)-2-(prop-2-yn-1-yl)hydrazine-1,2-dicarboxylate (5)[2]

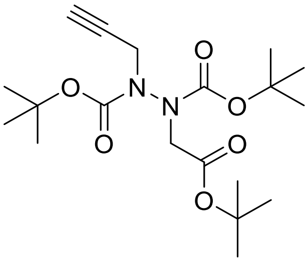

To a solution of di-*tert*-butyl 1-(prop-2-yn-1-yl)hydrazine-1,2-dicarboxylate (1.45 g, 5.36 mmol) in DMF (20 mL) were added caesium carbonate (3.56 g, 10.92 mmol) and then *tert*-butyl bromoacetate (1.57 g, 2.8 mmol), and the reaction mixture stirred at 20 °C for 16 h. After this time, the reaction mixture was diluted with H_2O (50 mL), extracted with EtOAc (4 × 50 mL), the combined organic layers washed with sat. aq. LiCl (2 × 30 mL), dried ($MgSO_4$), and concentrated *in vacuo*. Purification of the crude residue by flash column chromatography (10% EtOAc/petrol) yielded di-*tert*-butyl 1-(2-(*tert*-butoxy)-2-oxoethyl)-2-(prop-2-yn-1- yl)hydrazine-1,2-dicarboxylate **5** (2.00 g, 5.20 mmol, 97%) as a colourless oil: ^{1}H NMR (600 MHz, $CDCl_3$) δ 4.58–4.35 (m, 2H), 4.16–4.01 (m, 1H), 3.67–3.64 (m, 1H), 2.18 (t, J = 2.3 Hz, 1H), 1.53–1.41 (m, 27H); ^{13}C NMR (150 MHz, $CDCl_3$) (major rotamer) δ 168.1 (C), 166.3 (C), 153.9 (C), 83.0 (C), 81.9 (C), 81.8 (C), 79.4 (C), 71.9 (CH), 53.5 (CH_2), 40.1 (CH_2), 28.3 (CH_3), 28.3 (CH_3), 28.1 (CH_3); IR (thin film) 3265, 2108, 1714 cm^{-} ; LRMS (CI) 385 (55, $[M+H]^+$), 329 (65), 273 (100); HRMS (CI) calcd for $C_{19}H_{33}O_6N_2$ $[M+H]^+$ 385.2333, observed 385.2319.

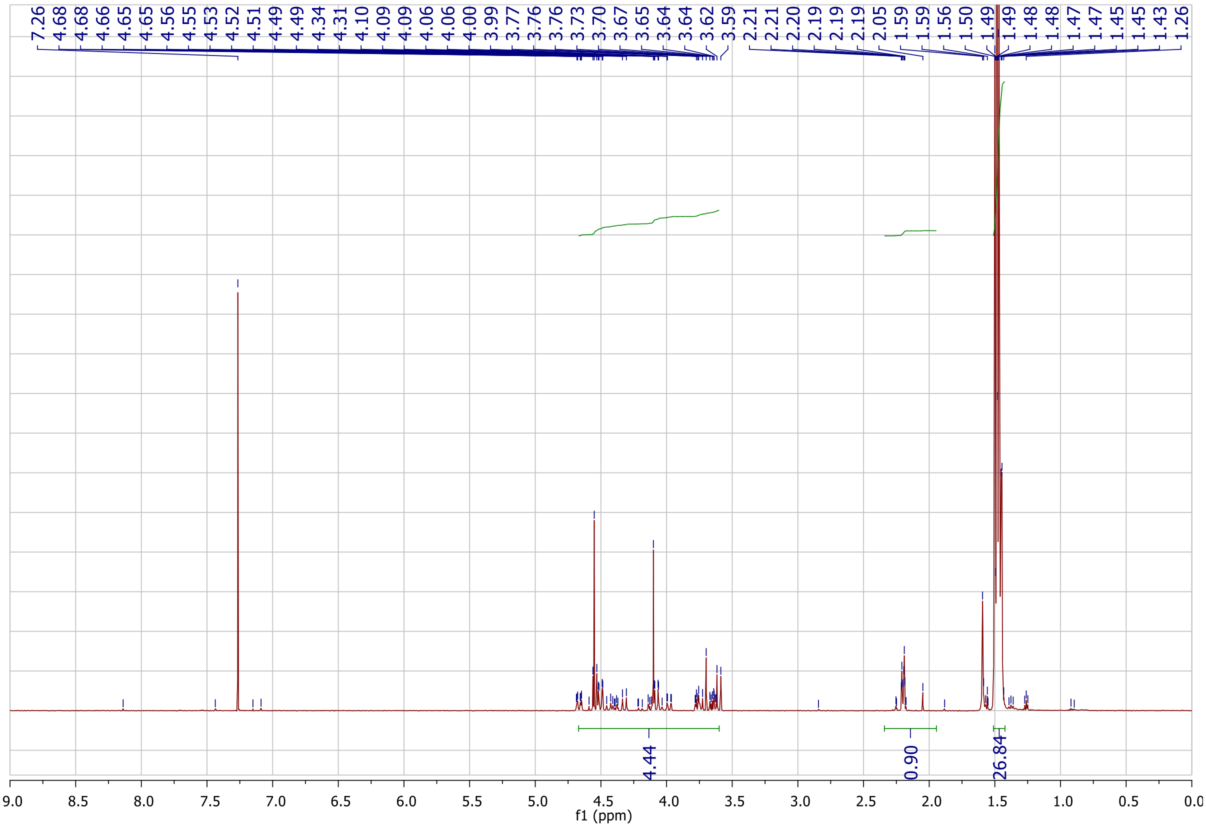

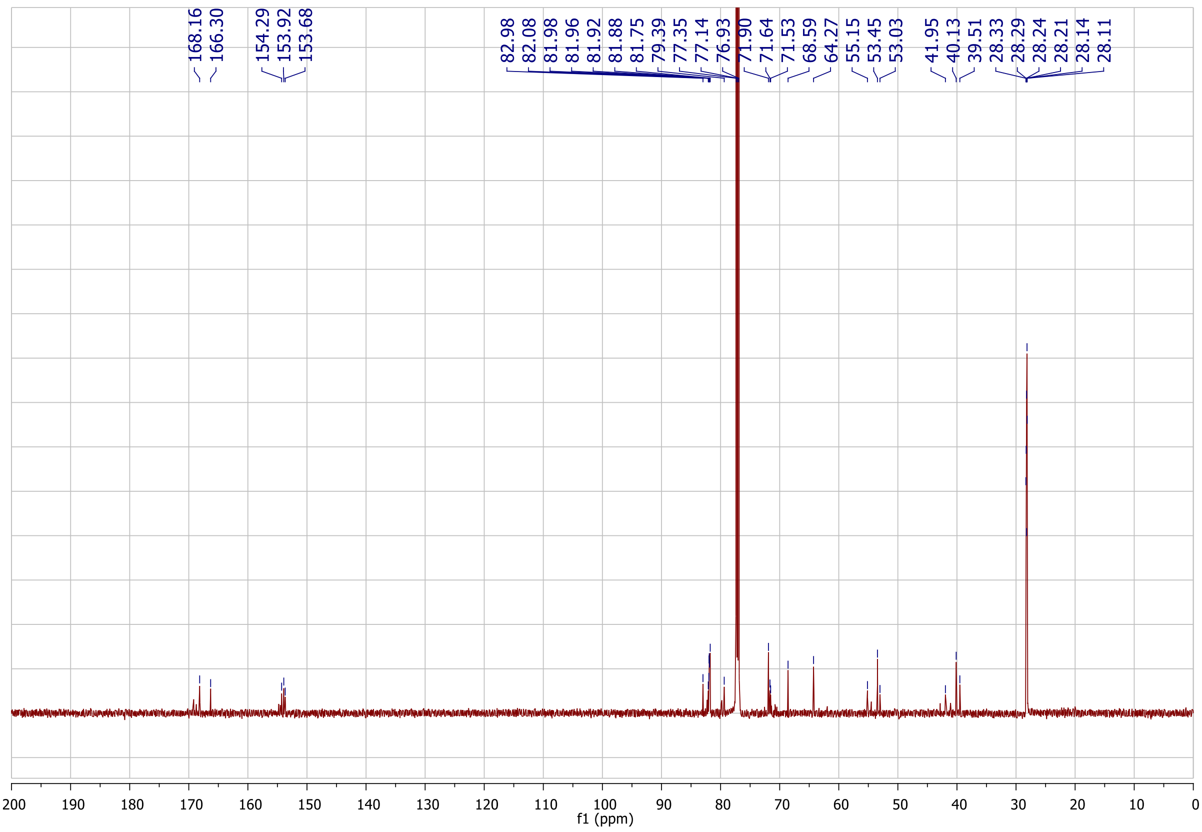

Figure **S6**. ^{1}H and ^{13}C NMR data for di-*tert*-butyl 1-(2-(*tert*-butoxy)-2-oxoethyl)-2-(prop-2-yn-1-yl)hydrazine-1,2-dicarboxylate **5**.

2-(4,5-Dibromo-3,6-dioxo-2-(prop-2-yn-1-yl)-2,3-dihydropyridazin-1(6*H*)-yl) acetic acid (6)[2]

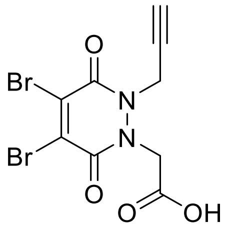

To a solution of di-*tert*-butyl 1-(2-(*tert*-butoxy)-2-oxoethyl)-2-(prop-2-yn-1-yl)hydrazine- 1,2-dicarboxylate **5** (0.50 g, 1.30 mmol) in CH_2Cl_2 (10 mL) was added TFA (10 mL), and the reaction mixture stirred at 20 °C for 2 h. After this time, all volatile materials were removed *in vacuo*. The crude residue was added to a solution of 2,3-dibromomaleic anhydride (0.40 g, 1.57 mmol) in glacial AcOH (40 mL), and the reaction mixture stirred at 20 °C for 16 h before raising the temperature to 130 °C for 2 h. Then the reaction mixture was concentrated *in vacuo*, and purification of the crude residue by flash column chromatography (3% MeOH/CH_2Cl_2 with 1% AcOH) yielded 2-(4,5-dibromo-3,6-dioxo-2-(prop-2-yn-1-yl)- 2,3-dihydropyridazin-1(6*H*)-yl)acetic acid **6** (0.31 g, 0.85 mmol, 65%) as a white solid: m.p. 108–110 °C; ^{1}H NMR (600 MHz, MeOD) δ 4.93 (s, 2H), 4.79 (d, *J* = 2.5 Hz, 2H), 2.46 (t, *J* = 2.5 Hz, 1H); ^{13}C NMR (150 MHz, $CDCl_3$) δ 168.1 (C), 153.9 (C), 152.6 (C), 136.3 (C), 135.9 (C), 77.3 (C), 75.4 (CH), 48.4 (CH_2), 37.1 (CH_2); IR (solid) 3444, 3287, 2109, 1729, 1631 cm^{-1}; LRMS (CI) 369 (50, $[M^{81}Br^{81}Br+H]^+$), 367 (100, $[M^{81}Br^{79}Br+H]^+$), 365 (50, $[M^{79}Br^{79}Br+H]^+$); HRMS (CI) calcd for $C_9H_7N_2O_4Br_2$ $[M^{79}Br^{79}Br+H]^+$ 364.8767, observed 364.8762.

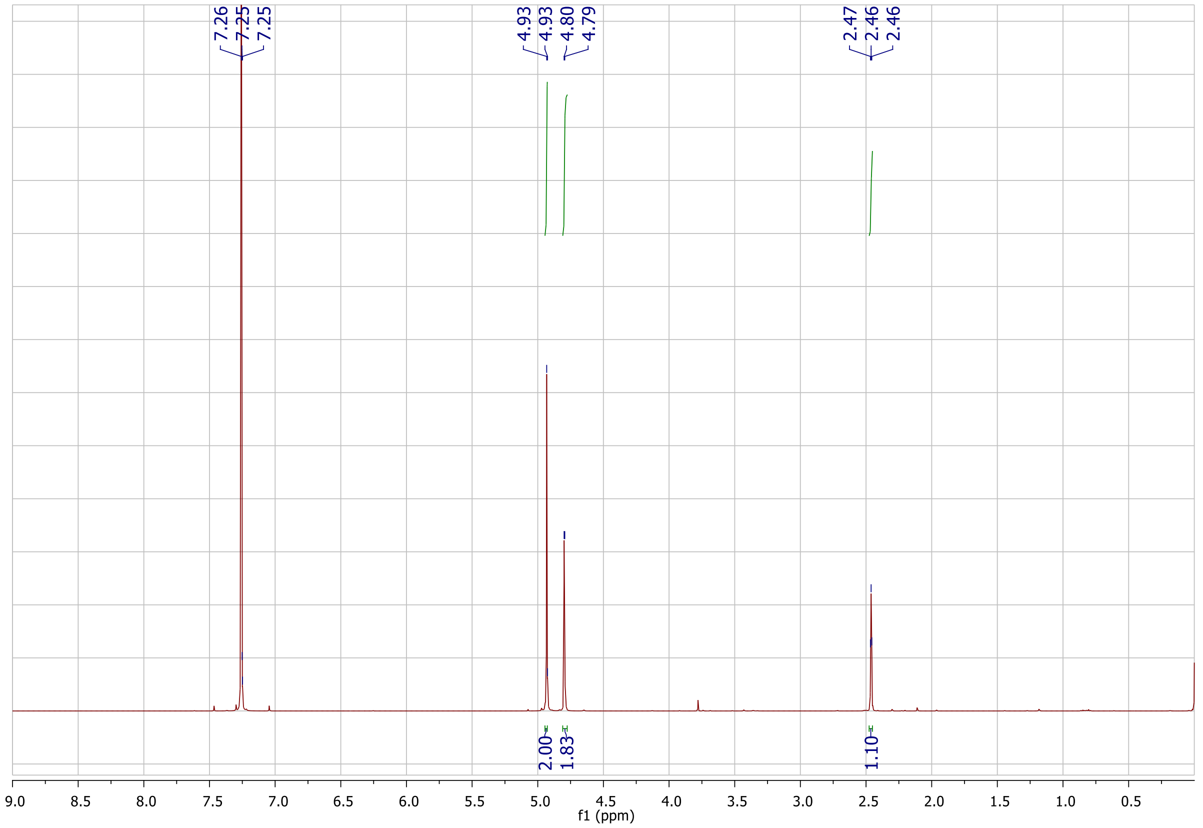

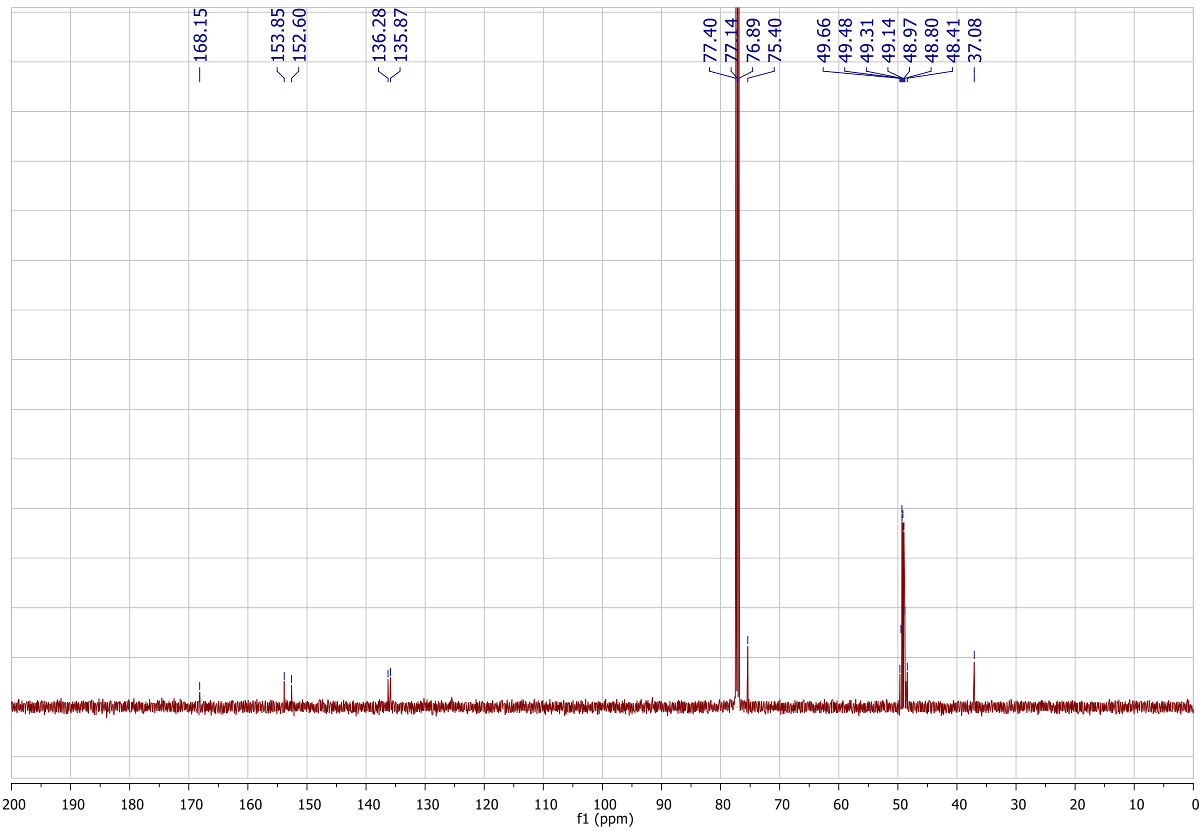

Figure **S7**. ^{1}H and ^{13}C NMR data for 2-(4,5-dibromo-3,6-dioxo-2-(prop-2-yn-1-yl)-2,3-dihydropyridazin-1(6*H*)-yl) acetic acid **6**.

tert-Butyl (3-(2-(2-(3-aminopropoxy)ethoxy)ethoxy)propyl)carbamate[3]

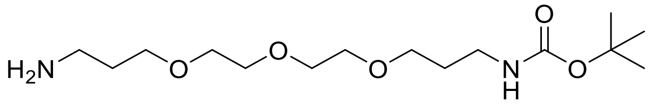

To a stirring solution of 3,3'-((oxybis(ethane-2,1-diyl))bis(oxy))bis(propan-1-amine) (8.10 g, 37.1 mmol) in 1,4-dioxane (60 mL) was added dropwise di-*tert*-butyl dicarbonate (1.00 g, 4.60 mmol, pre-dissolved in 1,4-dioxane (25 mL)) over 2 h, ensuring that the temperature did not exceed 21 °C. After this time, the reaction mixture was stirred at 21 °C for a further 30 mins. Following this, the reaction mixture was concentrated *in vacuo*, the crude residue dissolved in water (50 mL), and the organics extracted into EtOAc (5 × 30 mL). The organics were combined, dried ($MgSO_4$) and concentrated *in vacuo* to give *tert*-butyl (3-(2-(2-(3-aminopropoxy)ethoxy)ethoxy)propyl)carbamate (1.1 g, 34 mmol, 75%) as a colourless oil. ^{1}H NMR (600 MHz, $CDCl_3$) δ 5.11 (br s, 1H), 4.99 (d, *J* = 1.9 Hz, 4H), 4.93 (s, 4H), 3.65-3.61 (m, 12H), 3.24-3.19 (m, 2H), 2.79 (t, *J* = 6.7 Hz, 2H), 1.72 (tt, *J* = 6.5 Hz, 4H), 1.43 (s, 9H); ^{13}C NMR 151 MHz, $CDCl_3$) δ 156.2 (C), 79.0 (C), 70.7 (CH_2), 70.7 (CH_2), 70.4 (CH_2), 70.3 (CH_2), 69.7 (CH_2), 69.6 (CH_2), 39.8 (CH_2), 38.6 (CH_2), 33.5 (CH_2), 29.7 (CH_2), 28.6 (CH_3); IR (thin film) 3360, 2928, 2865, 1696, 1521, 1102 cm^{-1}.

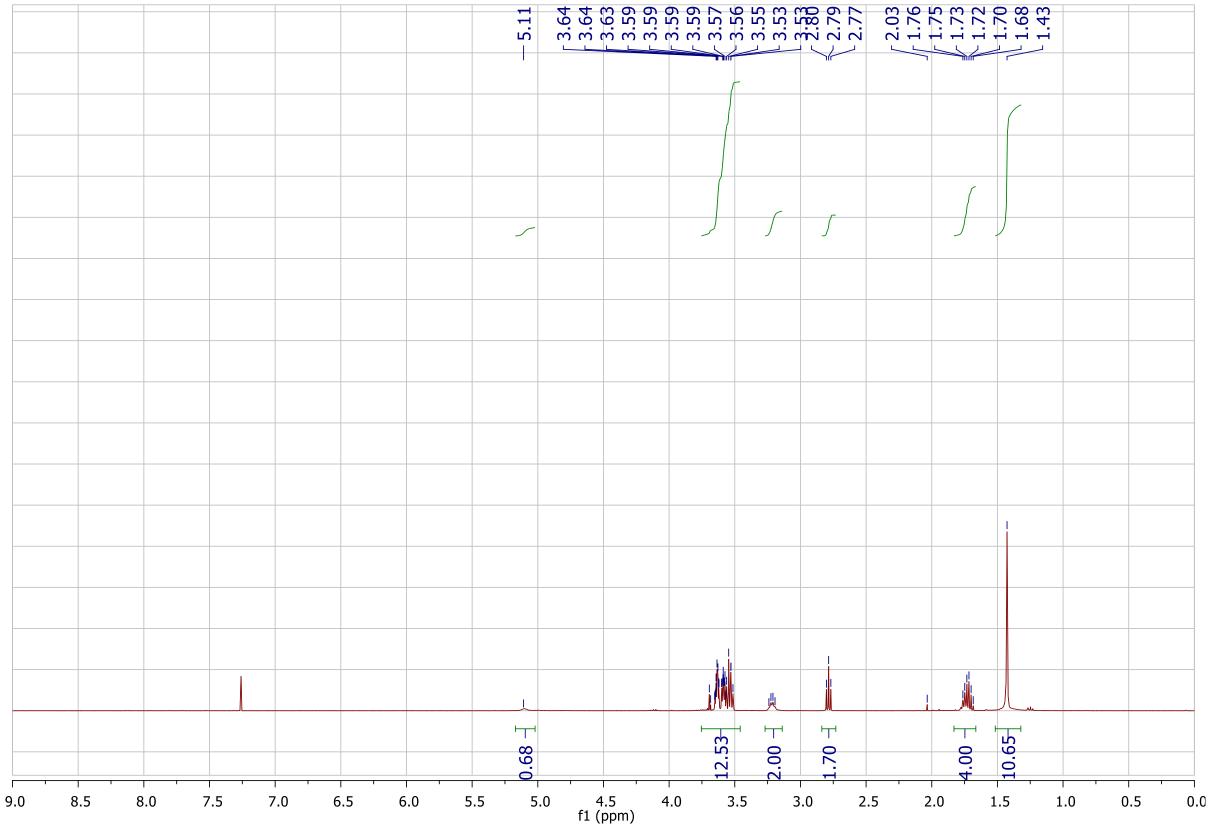

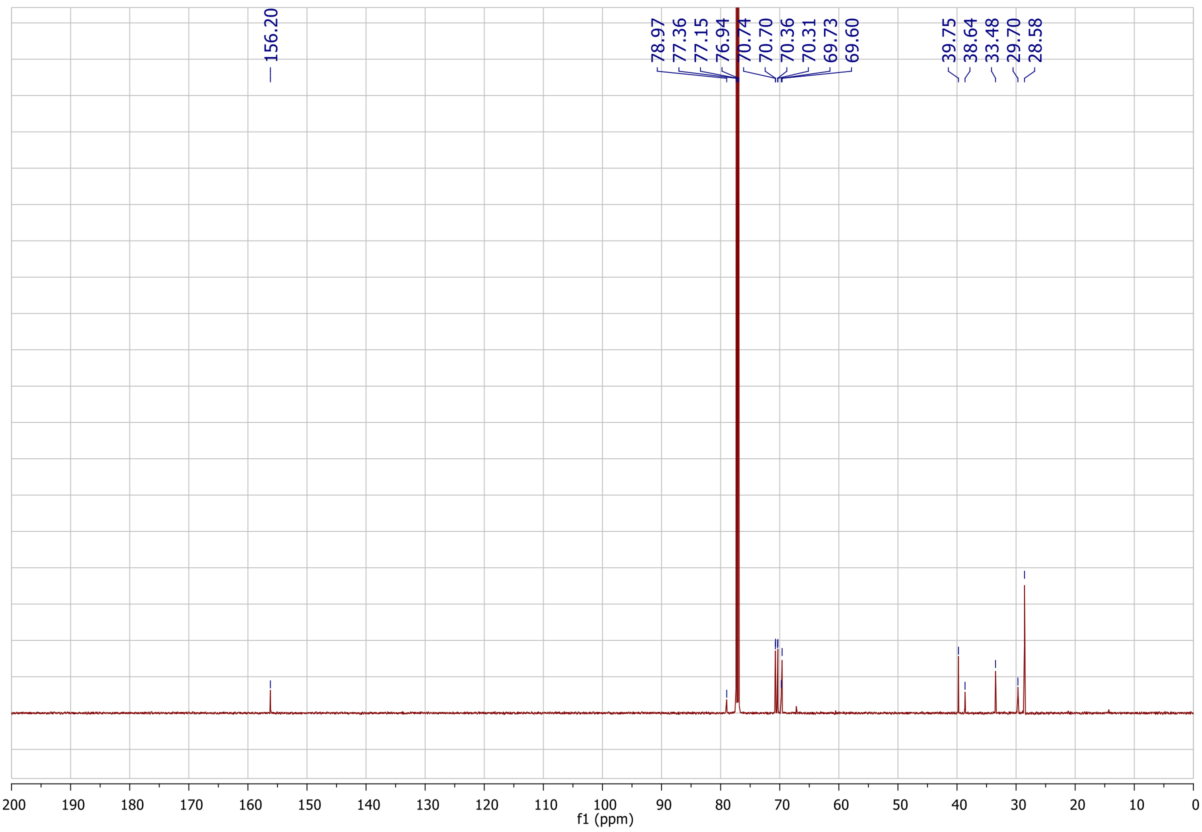

Figure **S8**. ^{1}H and ^{13}C NMR data for *tert*-butyl (3-(2-(2-(3-aminopropoxy)ethoxy)ethoxy)propyl)carbamate.

***tert*-Butyl (1-(4,5-dibromo-2-methyl-3,6-dioxo-3,6-dihydropyridazin-1(2*H*)-yl)-2-oxo-7,10,13-trioxa-3-azahexadecan-16-yl) (4)**

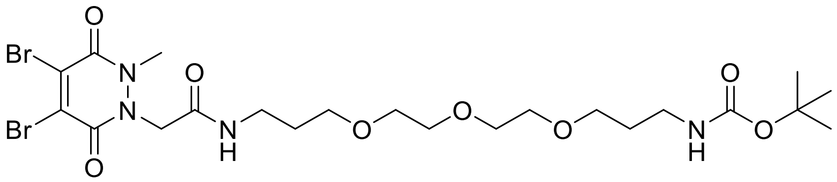

To a solution of 2-(4,5-dibromo-2-methyl-3,6-dioxo-3,6-dihydropyridazin-1(2*H*)-yl)acetic acid (350 mg, 1.02 mmol) in DMF (30 mL) was added CDI (165 mg, 1.02 mmol), and the reaction mixture stirred at 21 °C for 15 mins. After this time, to the reaction mixture was added *tert*-butyl (3-(2-(2-(3-aminopropoxy)ethoxy)ethoxy)propyl)carbamate) (362 mg, 1.13 mmol), and the mixture left to stir at 21 °C for a further 12 h. The reaction mixture was then diluted with EtOAc (50 mL) and washed with saturated sodium bicarbonate solution (1 × 20 mL), deionised water (4 × 20 mL) and brine (2 × 20 mL). The organic phase was then dried ($MgSO_4$) and concentrated *in vacuo*, and the resultant crude residue purified by flash column chromatography (0-20% MeOH:EtOAc). The appropriate fractions were combined and concentrated *in vacuo* to afford *tert*-butyl (1-(4,5-dibromo-2-methyl-3,6-dioxo-3,6-dihydropyridazin-1(2*H*)-yl)-2-oxo-7,10,13-trioxa-3-azahexadecan-16-yl)carbamate **4** (50 mg, 0.08 mmol, 8%) as a yellow gum. ^{1}H NMR (600 MHz, $CDCl_3$) δ 7.12 (br. s, 1H), 4.96 (br. s, 1H), 4.74 (s, 2H), 3.69 (s, 3H), 3.66-3.58 (m, 10H), 3.53 (t, *J* = 6.1 Hz, 2H), 3.42 (dd, *J* = 11.9, 5.7, 2H), 3.22-3.19 (m, 2H), 1.82-1.78 (m, 2H), 1.76-1.72 (m, 2H), 1.43 (s, 9H); ^{13}C NMR 151 MHz, $CDCl_3$) δ 165.3 (C), 156.2 (C), 153.5 (C), 152.5 (C), 136.9 (C), 134.9 (C), 79.2 (C), 70.5 (CH_2), 70.4 (CH_2), 70.2 (CH_2), 70.2 (CH_2), 69.9 (CH_2), 69.5 (CH_2), 50.7 (CH_2), 38.9 (CH_2), 38.4 (CH_2), 34.9 (CH_3), 29.9 (CH_2), 28.6 (CH_3), 28.5 (CH_2); IR (thin film) 3347, 2921, 2866, 1685, 1634, 1282, 1054 cm^{-1}; LRMS (ES+) 647 (50, $[M^{81}Br^{81}Br+H]^+$), 645 (100, $[M^{81}Br^{79}Br+H]^+$), 643 (50, $[M^{79}Br^{79}Br+H]^+$); HRMS (ES+) calcd for $C_{22}H_{37}Br_2N_4O_8$ $[M^{81}Br^{79}Br+H]^+$ 643.0973, observed 643.0954.

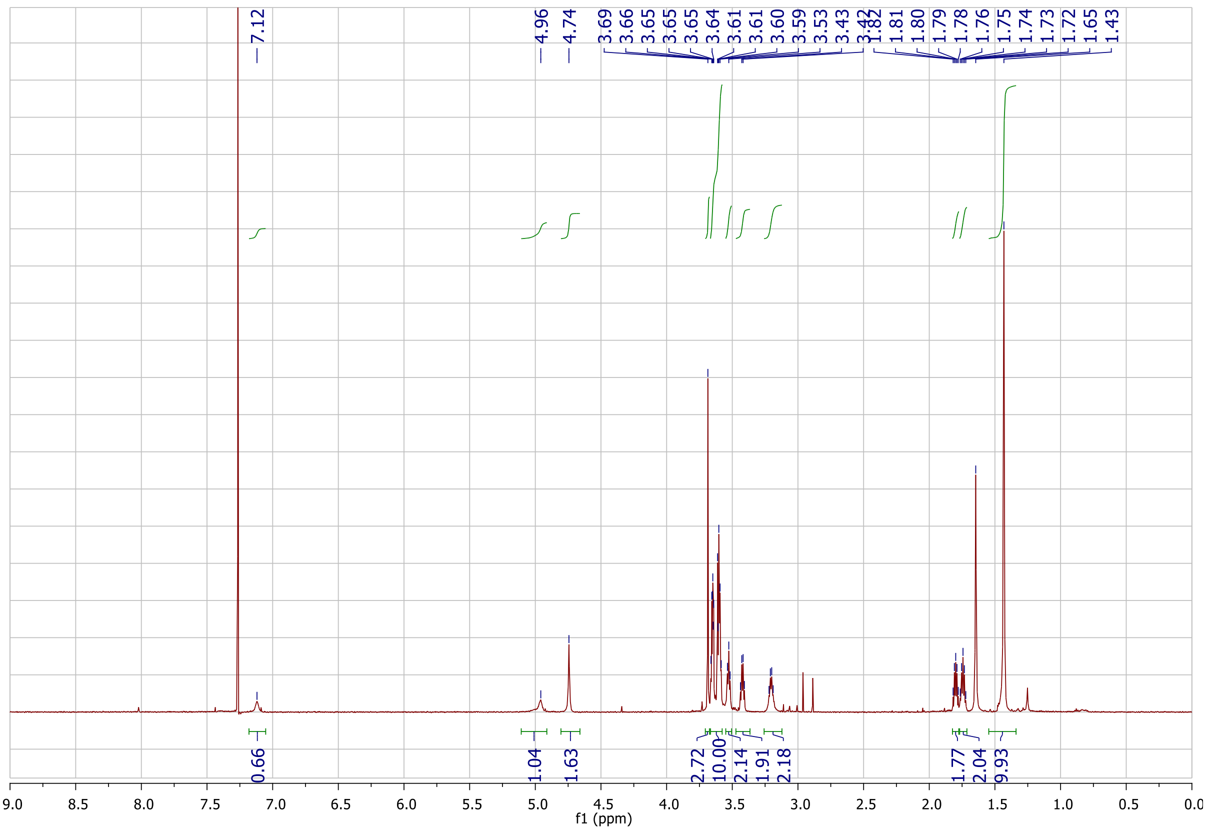

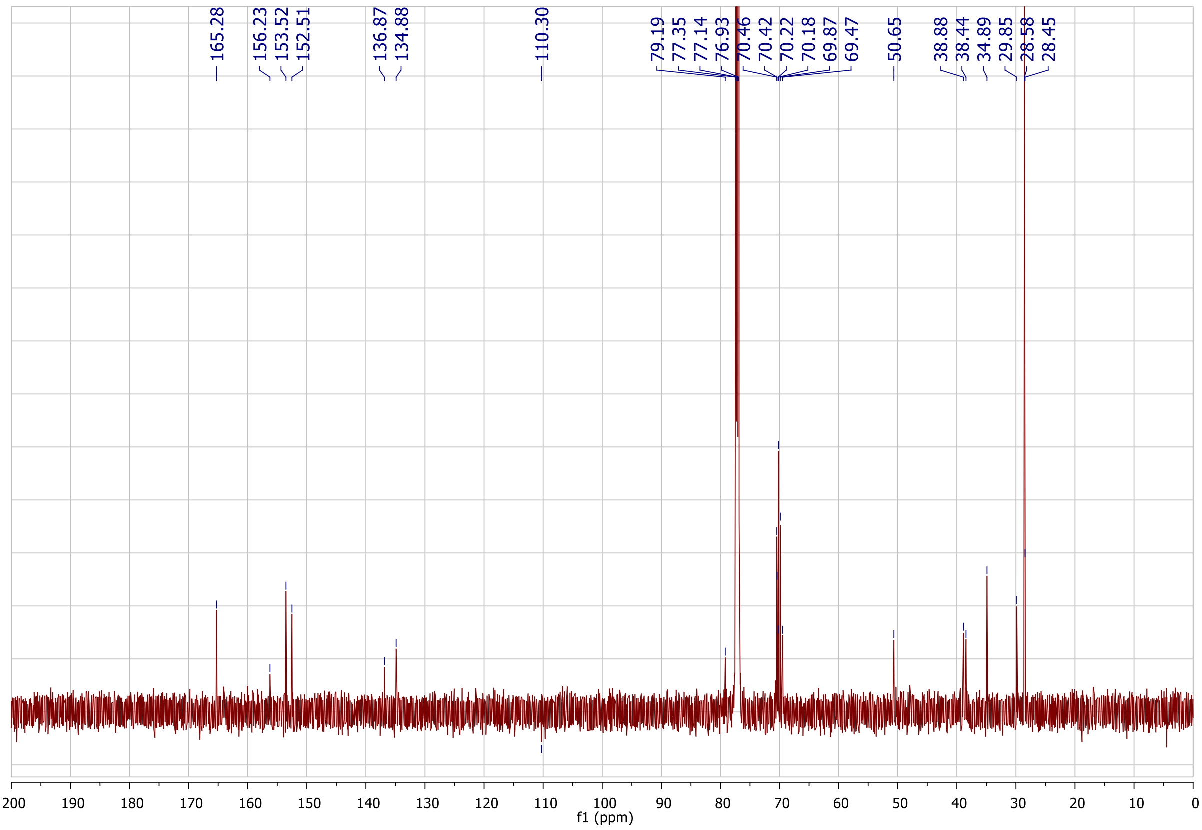

Figure **S9**. ^{1}H and ^{13}C NMR data for *tert*-butyl (1-(4,5-dibromo-2-methyl-3,6-dioxo-3,6-dihydropyridazin-1(2*H*)-yl)-2-oxo-7,10,13-trioxa-3-azahexadecan-16-yl)carbamate **4**.

2-(4,5-Dibromo-2-methyl-3,6-dioxo-3,6-dihydropyridazin-1(2*H*)-yl)-*N*-(1-(4,5-dibromo-3,6-dioxo-2-(prop-2-yn-1-yl)-3,6-dihydropyridazin-1(2*H*)-yl)-2-oxo-7,10,13-trioxa-3-azahexadecan-16-yl)acetamide (1)

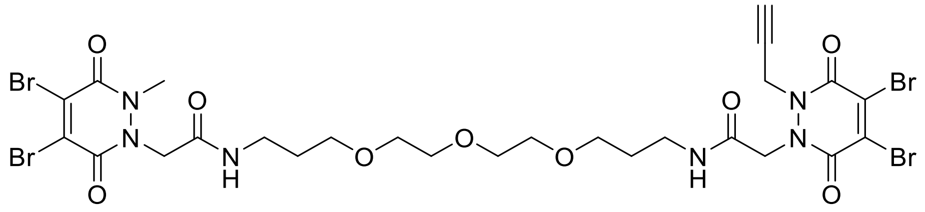

To a solution of 2-(4,5-dibromo-2-methyl-3,6-dioxo-3,6-dihydropyridazin-1(2*H*)-yl)-N-(1-(4,5-dibromo-3,6-dioxo-2-(prop-2-yn-1-yl)-3,6-dihydropyridazin-1(2*H*)-yl)-2-oxo-7,10,13-trioxa-3-azahexadecan-16-yl)acetamide (50.0 mg, 0.08 mmol) in CH_2Cl_2 (5 mL) was added TFA (5 mL), and the reaction mixture stirred for 30 mins at 21 °C. After this time, the reaction mixture was concentrated *in vacuo* and the residue dissolved in CH_2Cl_2 (5 mL). The resultant solution was added to a pre-mixed (20 mins at 21 °C) solution of 2-(4,5-dibromo-3,6-dioxo-2-(prop-2-yn-1-yl)-3,6-dihydropyridazin-1(2*H*)-yl)acetic acid (27.3 mg, 0.08 mmol), HATU (28.4 mg, 0.08 mmol) and *N*-ethyldiisopropylamine (21.2 mg, 0.16 mmol, 30 µL) in CH_2Cl_2 (10 mL). The reaction mixture was then stirred for 16 h at 21 °C. Following this, the reaction mixture was washed with water (3 × 10 mL) and the resultant aqueous washes extracted with CH_2Cl_2 (10 mL). The organic phases were combined, dried ($MgSO_4$) and concentrated *in vacuo*, and the crude residue purified by flash column chromatography (0-15% MeOH:EtOAc). The appropriate fractions were combined and concentrated *in vacuo* to afford 2-(4,5-dibromo-2-methyl-3,6-dioxo-3,6-dihydropyridazin-1(2*H*)-yl)-*N*-(1-(4,5-dibromo-3,6-dioxo-2-(prop-2-yn-1-yl)-3,6-dihydropyridazin-1(2*H*)-yl)-2-oxo-7,10,13-trioxa-3-azahexadecan-16-yl)acetamide **1** (20 mg, 0.02 mmol, 30%) as a yellow gum. ^{1}H NMR (600 MHz, $CDCl_3$) δ 7.25 (t, *J* = 5.4 Hz, 1H), 7.21 (t, *J* = 5.2 Hz, 1H), 4.99 (d, *J* = 2.0 Hz, 2H), 4.93 (s, 2H), 4.79 (s, 2H), 3.68 (s, 3H), 3.67-3.57 (m, 14H), 3.40 (td, *J* = 12.3, 5.9, 4H), 2.46 (t, *J* = 2.0 Hz, 1H), 1.82-1.76 (m, 4H); ^{13}C NMR 151 MHz, $CDCl_3$) δ 165.4 (C), 165.3 (C), 153.7 (C), 153.6 (C), 152.5 (C), 152.4 (C), 137.0 (C), 136.6 (C), 135.8 (C), 134.8 (C), 76.3 (C), 75.0 (CH), 70.3 (CH_2), 69.8 (CH_2), 69.7 (CH_2), 69.6 (CH_2), 69.6 (CH_2), 50.6 (CH_2), 50.5 (CH_2), 38.4 (CH_2), 38.3 (CH_2), 37.4 (CH_2), 34.9 (CH_3), 28.5 (CH_2), 28.5 (CH_2); IR (thin film) 3305, 2924, 2870, 1636, 1571, 1287 cm^{-1}; LRMS (ES+) 895 (55, [M^{81}Br^{81}Br^{81}Br^{79}Br +H]$^+$), 893 (100, [M^{81}Br^{81}Br^{79}Br^{79}Br+H]$^+$), 891 (55, [M^{81}Br^{79}Br ^{79}Br^{79}Br+H]$^+$); HRMS (ES+) calcd for $C_{28}H_{32}Br_4N_6O_9$ [M^{79}Br^{79}Br^{79}Br^{79}Br+H]$^+$ 888.9037, observed 888.9039.

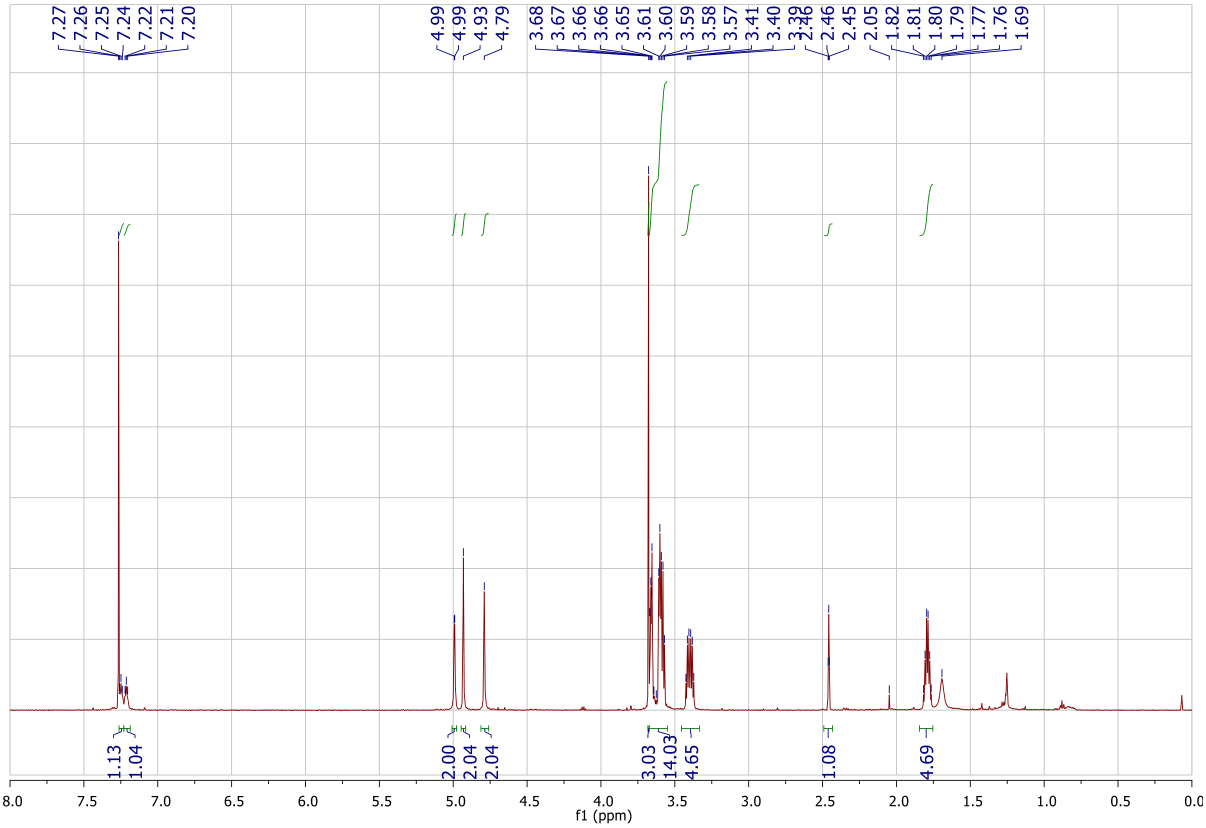

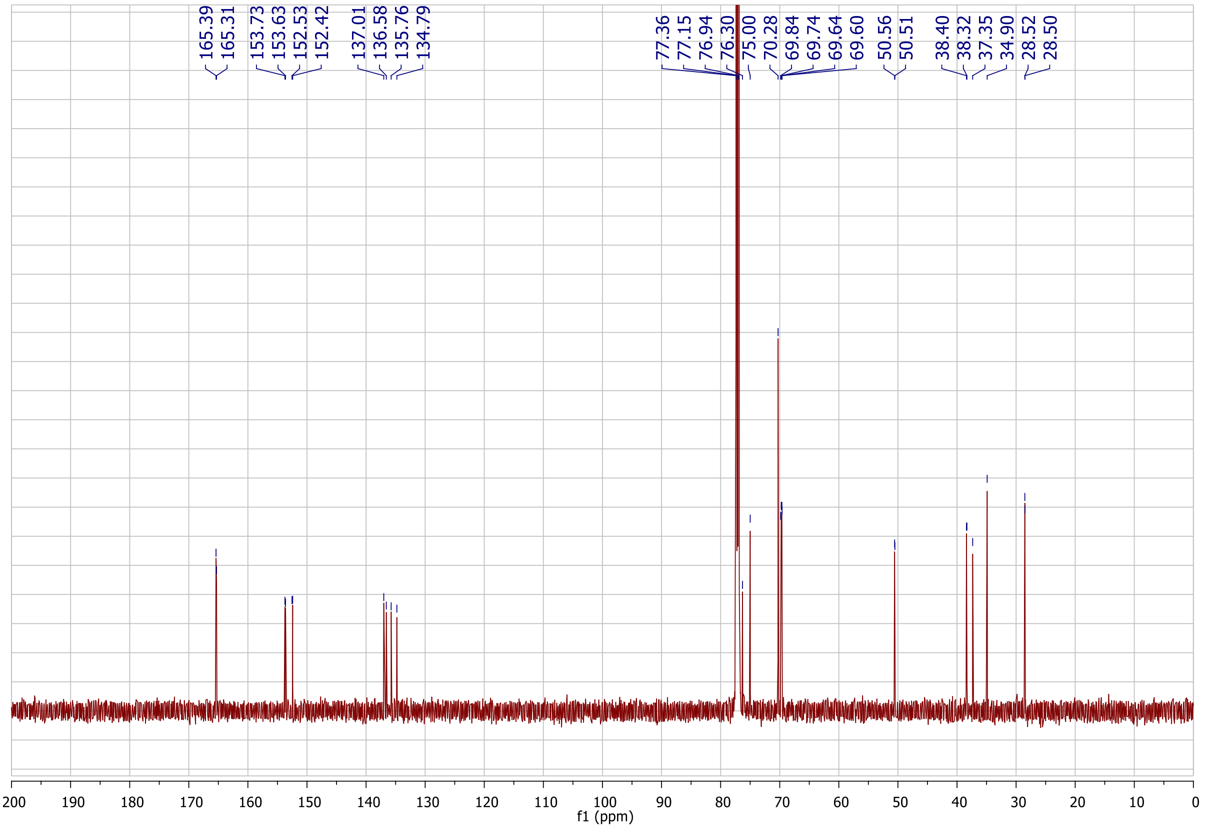

Figure **S10**. ^{1}H and ^{13}C NMR data for 2-(4,5-dibromo-2-methyl-3,6-dioxo-3,6-dihydropyridazin-1(2*H*)-yl)-*N*-(1-(4,5-dibromo-3,6-dioxo-2-(prop-2-yn-1-yl)-3,6-dihydropyridazin-1(2*H*)-yl)-2-oxo-7,10,13-trioxa-3-azahexadecan-16-yl)acetamide **1**.

***N*,*N'*-(((Oxybis(ethane-2,1-diyl))bis(oxy))bis(propane-3,1-diyl))bis(2-(4,5-dibromo-3,6-dioxo-2-(prop-2-yn-1-yl)-3,6-dihydropyridazin-1(2*H*)-yl)acetamide) (9)**

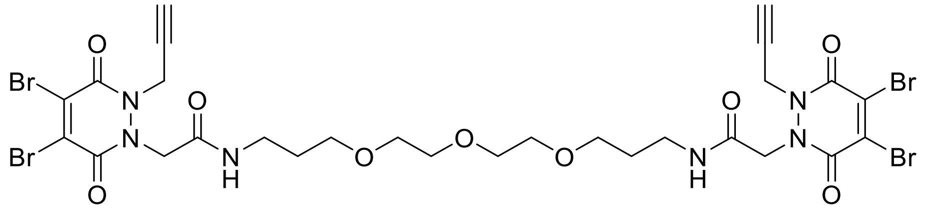

To a solution of 2-(4,5-dibromo-3,6-dioxo-2-(prop-2-yn-1-yl)-3,6-dihydropyridazin-1(2*H*)-yl)acetic acid (200 mg, 0.60 mmol) in DMF (5 mL) was added DCC (120 mg, 0.58 mmol), and the reaction mixture stirred at 21 °C for 15 mins. After this time, to the reaction mixture was added 3,3'-((oxybis(ethane-2,1-diyl))bis(oxy))bis(propan-1-amine) (26 µL, 0.12 mmol), and the resultant mixture stirred at 21 °C for 12 h. After this time, the reaction mixture was diluted with EtOAc (20 mL) and washed with deionised water (4 × 10 mL) and brine (2 × 10 mL). The organic phase was then dried ($MgSO_4$), concentrated *in vacuo* and the crude residue purified by flash column chromatography (0-10% MeOH:CH_2Cl_2), which yielded *N*,*N*'-(((oxybis(ethane-2,1-diyl))bis(oxy))bis(propane-3,1-diyl))bis(2-(4,5-dibromo-3,6-dioxo-2-(prop-2-yn-1-yl)-3,6-dihydropyridazin-1(2*H*)-yl)acetamide) (20 mg, 0.02 mmol, 18%) as a yellow gum. ^{1}H NMR (600 MHz, $CDCl_3$) δ 7.22 (t, *J* = 5.4 Hz, 2H), 4.99 (d, *J* = 1.9 Hz, 4H), 4.93 (s, 4H), 4.99 (d, *J* = 1.9 Hz, 4H), 3.68–3.62 (m, 4H), 3.62–3.56 (m, 8H), 3.39 (dd, *J* = 12.1, 5.9, 4H), 2.46 (t, *J* = 2.4 Hz, 2H), 1.79 (dt, *J* = 12.1, 5.9, 4H); ^{13}C NMR 151 MHz, $CDCl_3$) δ 165.4 (C), 153.8 (C), 152.4 (C), 136.5 (C), 135.8 (C), 76.3 (C), 75.0 (CH), 70.3 (CH_2), 69.8 (CH_2), 69.7 (CH_2), 50.5 (CH_2), 38.4 (CH_2), 37.3 (CH_2), 28.5 (CH_2); IR (thin film) 3299, 2928, 2052, 1643, 1285 cm^{-1}; LRMS (ES+) 917 (50, $[M^{81}Br^{81}Br^{81}Br^{79}Br+H]^+$), 915 (100, $[M^{81}Br^{81}Br^{79}Br^{79}Br+H]^+$), 913 (50, $[M^{81}Br^{79}Br\ ^{79}Br^{79}Br+H]^+$); HRMS (ES+) calcd for $C_{28}H_{32}Br_4N_6O_9$ $[M^{81}Br^{79}Br\ ^{79}Br^{79}Br+H\ ]^+$ 912.9042, observed 912.9048.

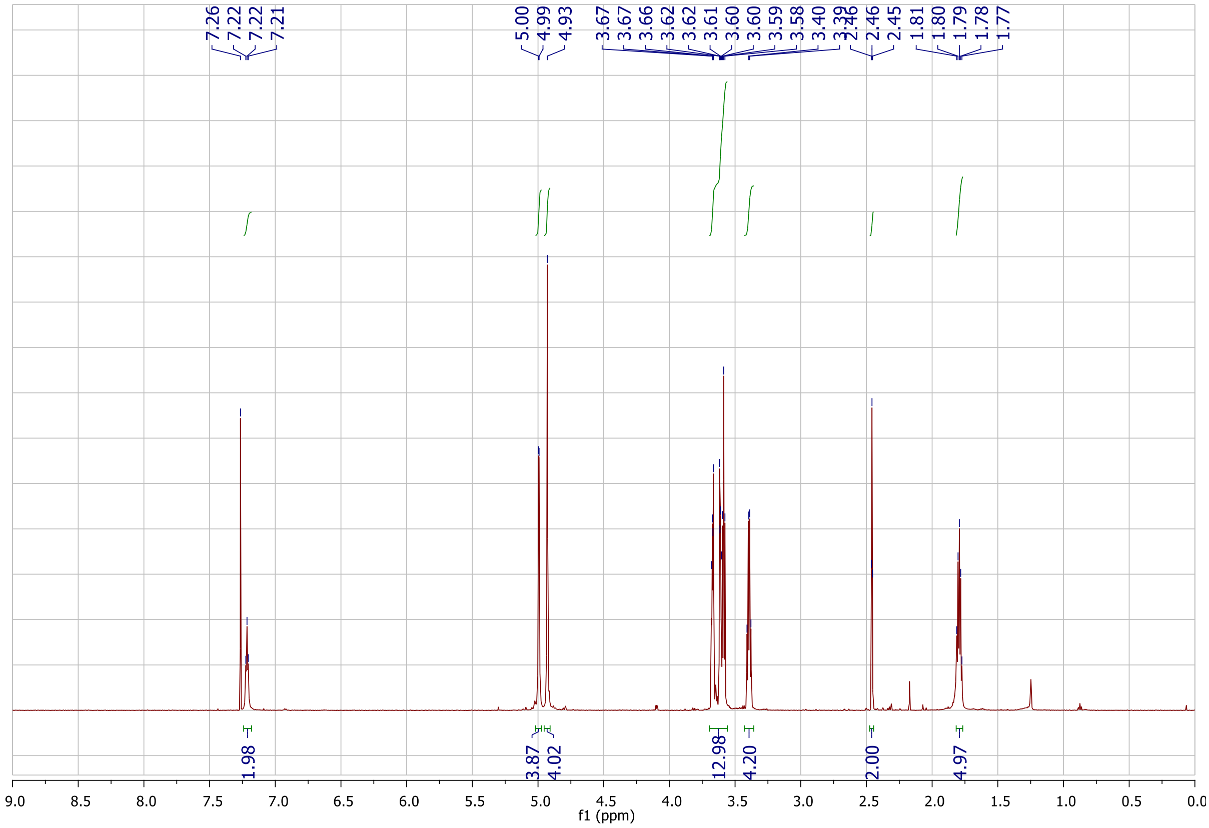

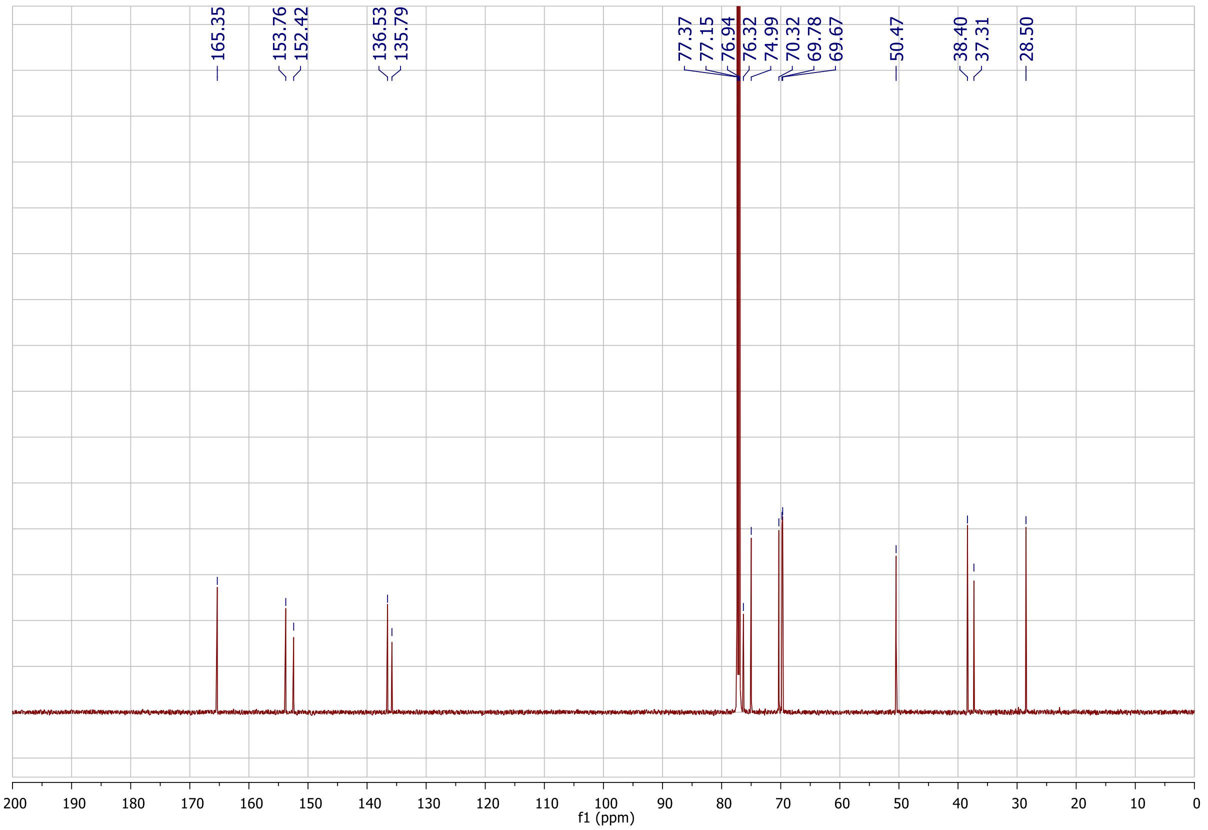

Figure **S11**. ^{1}H and ^{13}C NMR data for ’,*N*'-(((Oxybis(ethane-2,1-diyl))bis(oxy))bis(propane-3,1-diyl))bis(2-(4,5-dibromo-3,6-dioxo-2-(prop-2-yn-1-yl)-3,6-dihydropyridazin-1(2*H*)-yl)acetamide) **9**.

Di-*tert*-butyl 1,2-diethylhydrazine-1,2-dicarboxylate[4]

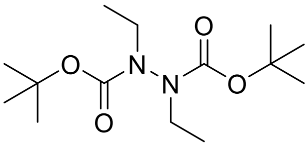

To a solution of di-*tert*-butyl hydrazine-1,2-dicarboxylate (1.0 g, 4.3 mmol) and caesium carbonate (5.6 g, 17.2 mmol) in DMF (20 mL) was added bromoethane (1.1 g, 10.1 mmol) and the reaction mixture stirred at 21 °C for 24 h. After this time, the reaction mixture was diluted with EtOAc (50 mL) and washed with deionised water (4 × 20 mL) and brine (2 × 20 mL). The organic phase was then dried ($MgSO_4$) and concentrated *in vacuo* and the crude residue purified by flash column chromatography (50% EtOAc:Pet.). The appropriate fractions were combined and concentrated *in vacuo* to afford di-*tert*-butyl 1,2-diethylhydrazine-1,2-dicarboxylate (1.1 g, 3.8 mmol, 88%) as a colourless oil as a mixture of rotamers. ^{1}H NMR (300 MHz, $CDCl_3$) δ 3.57–3.33 (m, 4H), 1.50–1.39 (m, 18H), 1.16 (t, J = 7.2 Hz, 6H); ^{13}C NMR (75 MHz, $CDCl_3$) δ 155.1 (C), 80.6 (C), 46.4 (CH_2), 44.4 (CH_2), 28.4 (CH_3), 28.3 (CH_3), 13.6 (CH_3), 13.0 (CH_3); IR (thin film) 2980, 2927, 2881, 1698 cm^{-1}; LRMS (ES+) 289 (100, $[M+H]^+$)

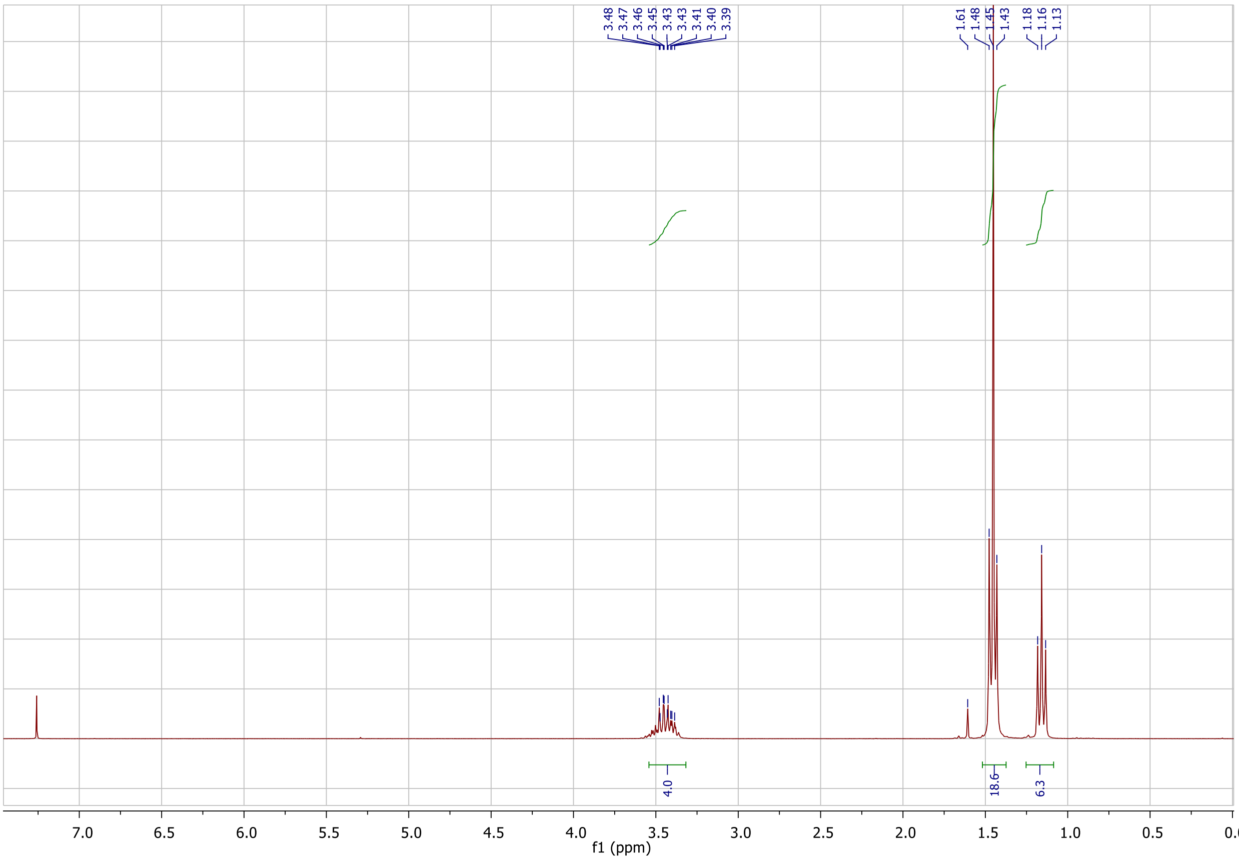

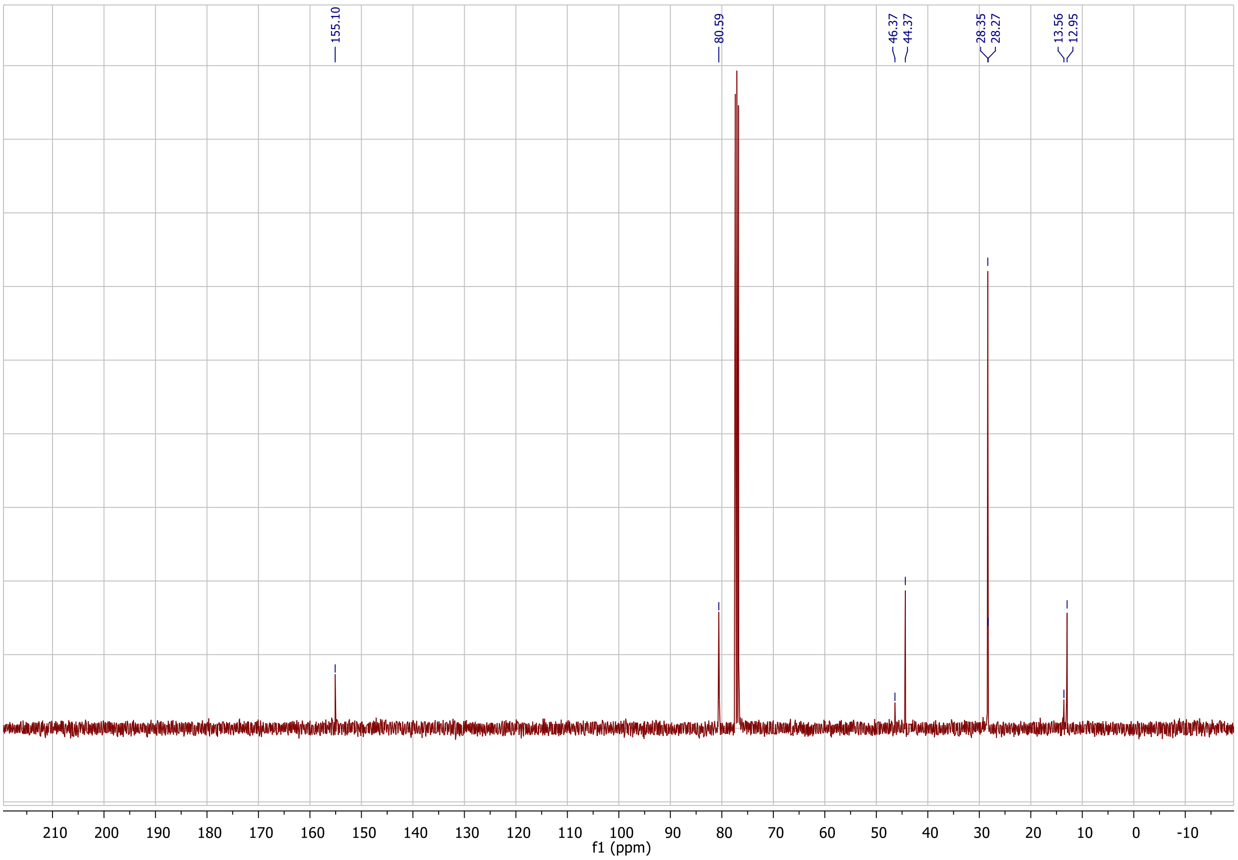

Figure **S12**. ^{1}H and ^{13}C NMR data for di-*tert*-butyl 1,2-diethylhydrazine-1,2-dicarboxylate.[4]

4,5-Dibromo-1,2-diethyl-1,2-dihydropyridazine-3,6-dione (DiEt PD)[4]

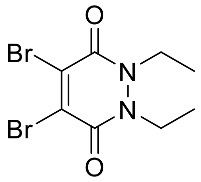

To a 1:1 solution of CH_2Cl_2:TFA (5 mL:5 mL) was added di-*tert*-butyl 1,2-diethylhydrazine-1,2-dicarboxylate (348 mg, 1.21 mmol) and the reaction mixture stirred at 21 °C for 1 h. The reaction mixture was then concentrated *in vacuo*, taking care to ensure all the TFA was removed by use of toluene to form an azeotrope. The crude residue was then dissolved in AcOH (10 mL), to which was added 3,4-dibromomaleic anhydride (310 mg, 1.21 mmol) and the reaction mixture heated under reflux for 2 h. The reaction mixture was then concentrated *in vacuo* and the crude residue was purified by flash column chromatography (30–50 % EtOAc:Pet.). The appropriate fractions were then combined and concentrated *in vacuo* to afford 4,5-dibromo-1,2-diethyl-1,2-dihydropyridazine-3,6-dione (DiEt PD) (288 mg, 0.88 mmol, 73%) as a pale yellow solid. ^{1}H NMR (600 MHz, $CDCl_3$) δ 4.17 (q, *J* = 7.0 Hz, 4H), 1.28 (t, *J* = 7.0 Hz, 6H); ^{13}C NMR (150 MHz, $CDCl_3$) δ 153.3 (C), 136.1 (C), 42.4 (CH_2), 13.2 (CH_3); IR (solid) 2979, 2937, 1630, 1574 cm^{-1}; LRMS (EI) 328 (50, [$M^{81}Br^{81}Br$]$^+$), 326 (100, [$M^{81}Br^{79}Br$]$^+$), 324 (50, [$M^{79}Br^{79}Br$]$^+$); HRMS (EI) calcd for $C_8H_{10}Br_2N_2O_2$ [$M^{79}Br^{79}Br$]$^+$ 323.9104, observed 323.9097.

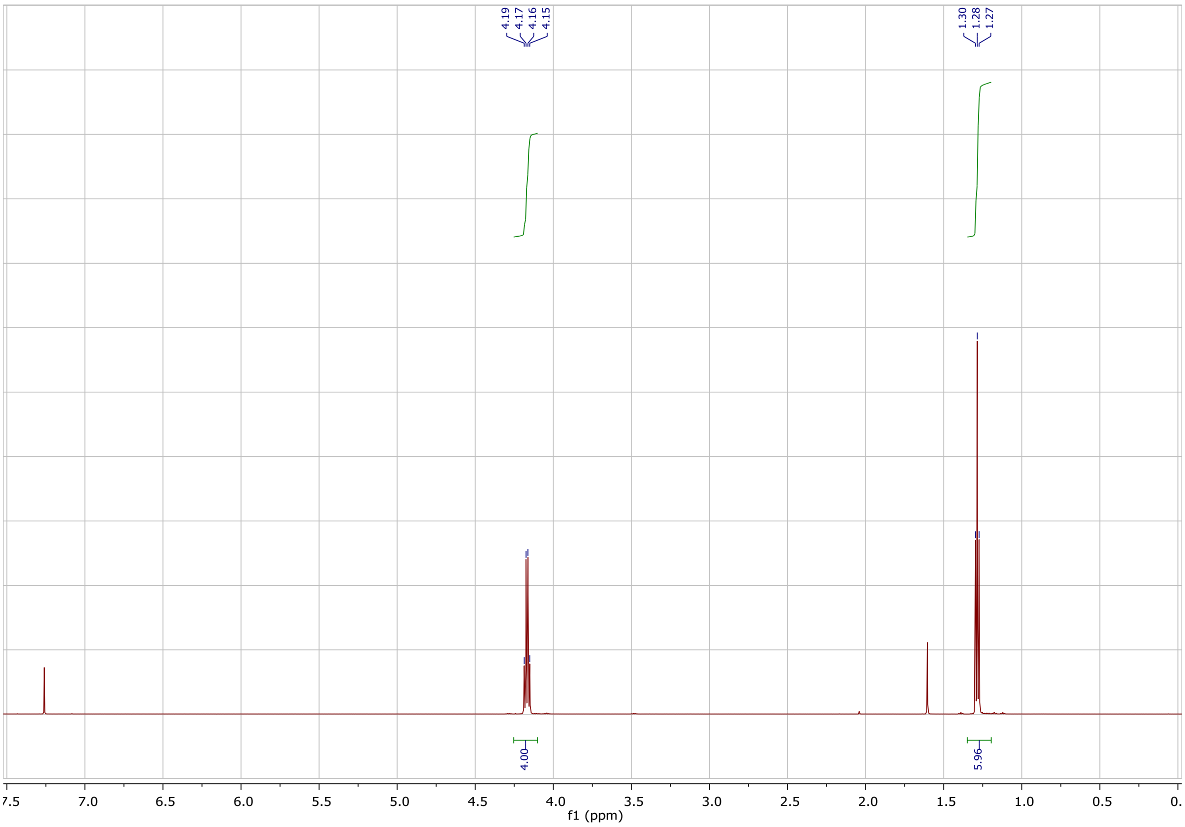

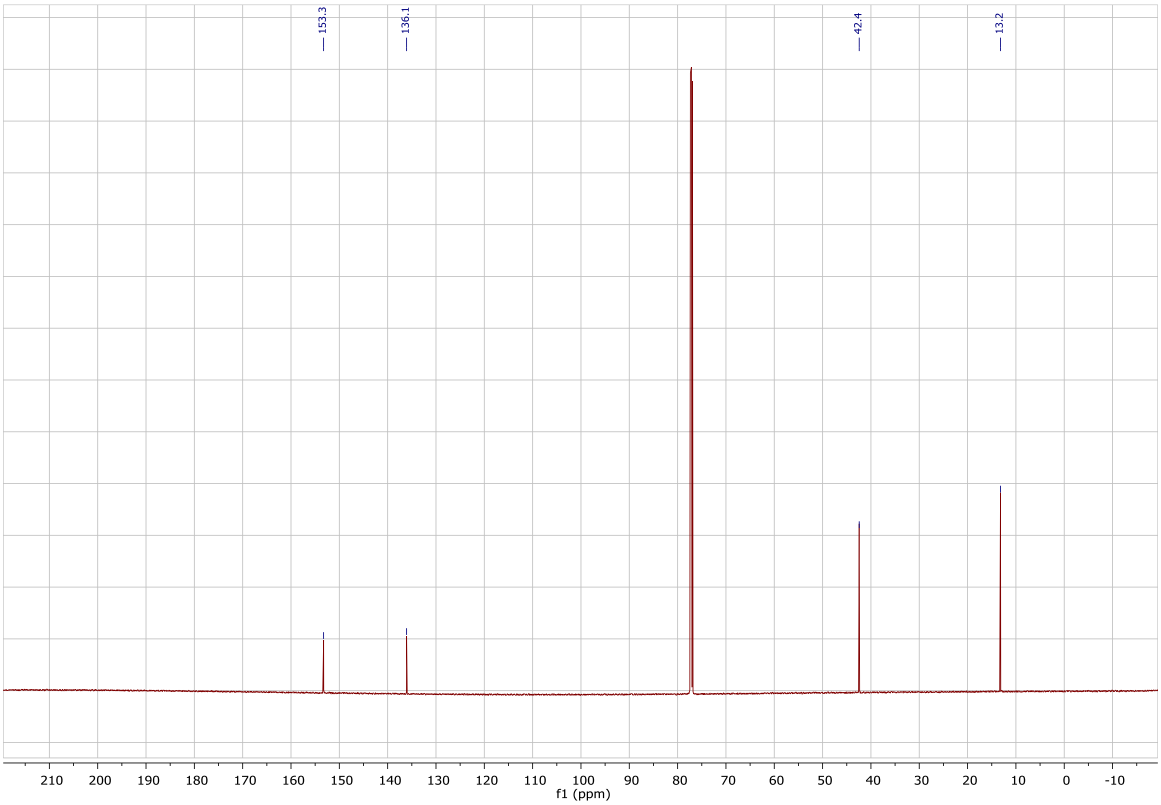

Figure **S13**. ^{1}H and ^{13}C NMR data for 4,5-dibromo-1,2-diethyl-1,2-dihydropyridazine-3,6-dione (DiEt PD).[4]

In situ reduction of Herceptin™ mAb and reaction with bis-diBrPD 1 at 4 °C (16 eq.) for the formation of conjugate 7

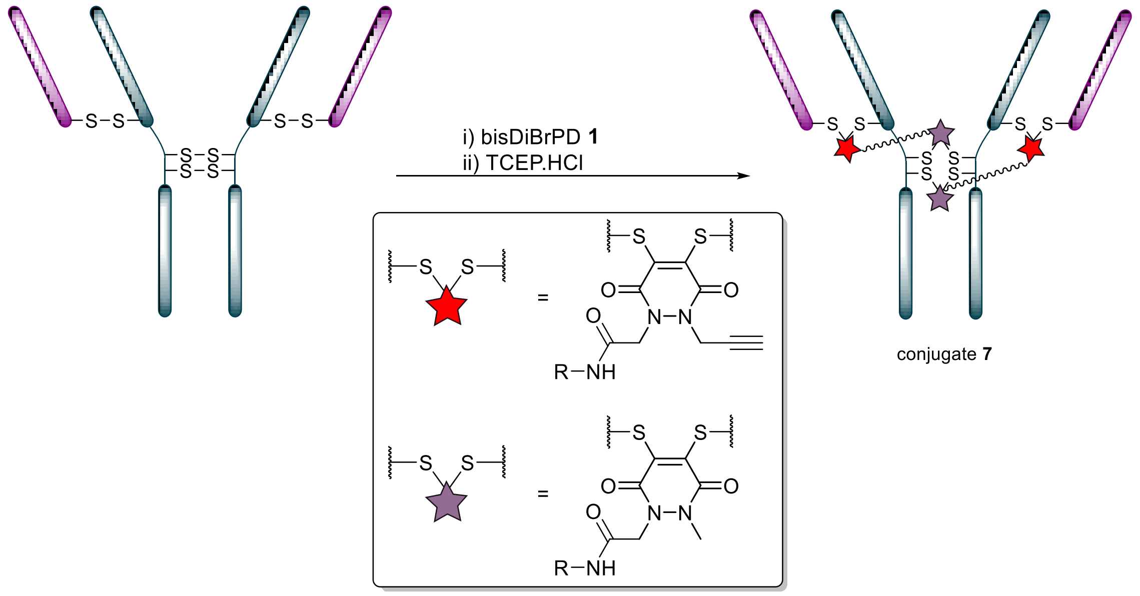

Each pair of red and purple stars per linker molecule are independently interchangeable, and other disulfide pairs (e.g. Fab-Fab, Hinge-Hinge) may be functionally re-bridged.

TCEP.HCl (10 µL, 20 mM in deionised water, 80 eq.) was added to a solution of Herceptin™ (50 µL, 7.5 mg/mL, 50 µM) in BBS (25 mM sodium borate, 25 mM NaCl, 0.5 mM EDTA, pH 8.0) which had been pre-treated with bis-diBrPD **1** (2.0 µL, 20 mM in DMSO, 16 eq.) and stored at 4 °C for 1 h previously. The reaction mixture was then stored at 4 °C for 15 h. Excess reagents were removed by repeated diafiltration into deionised water using VivaSpin sample concentrators (GE Healthcare, 10,000 MWCO). The samples were analysed by SDS-PAGE gel and UV-Vis spectroscopy, which was used to determine a PDAR of 4.0. This reaction was repeated on 7 separate occasions with remarkable reproducibility; PDARs in the range of 3.9-4.1 were observed. A TCEP reduction control was also conducted on conjugate **7**: TCEP.HCl (10 µL, 20 mM in deionised water, 80 eq.) was added to conjugate **7** (50 µL, 7.5 mg/mL, 50 µM) in BBS (25 mM sodium borate, 25 mM NaCl, 0.5 mM EDTA, pH 8.0), the reaction mixture was then incubated at 37 °C for 1 h. The sample was then analysed by LCMS following deglycosylation. Expected mass: 146,320 Da. Observed mass: 146,328 Da. Whilst there were some adducts, none of these corresponded to 3 or 4 additions of bis-diBrPD **1**. The sample was analysed further by SDS-PAGE gel (lane 3, below). As there was no fragmentation observed (going from lane 2 to lane 3) it was concluded that no accessible inter-chain disulfide bonds were present in conjugate **7** (control Herceptin™ reduction below, Figure S20). This experiment, combined with the lack of any fragments being observed in the SDS-PAGE gel in lane 2+3 and the UV-Vis data, confirmed that all accessible disulfides bonds were functionally re-bridged with PD molecules.

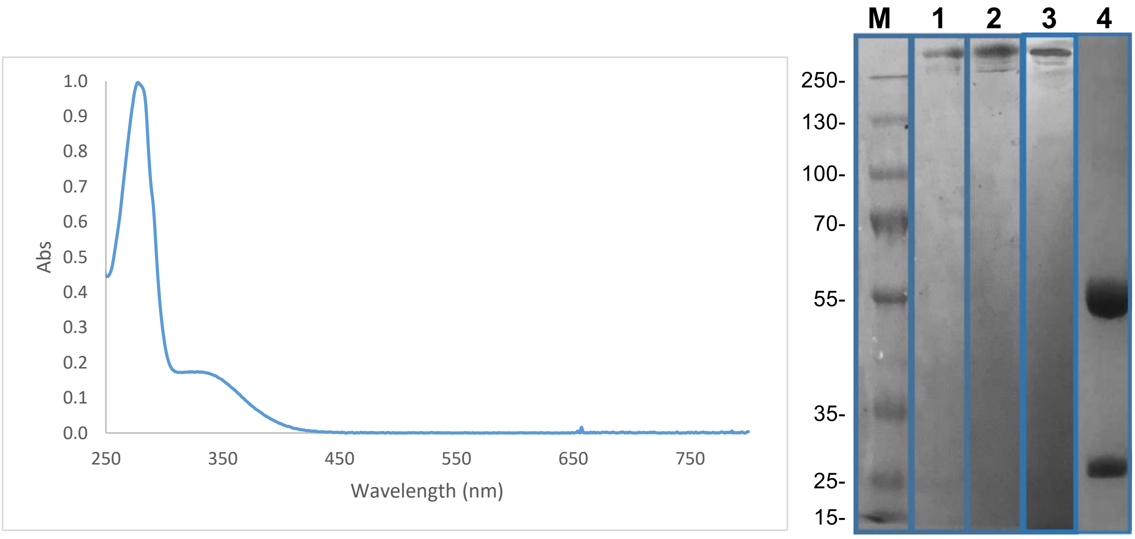

Figure **S14a**. (Left) UV-Vis data for conjugate **7**. (Right) SDS-PAGE gel; M.) Molecular weight marker; 1) Unmodified Herceptin™; 2) Conjugate **7**; 3) Conjugate **7** treated with TCEP.HCl; 4) Herceptin™ reduced with TCEP.HCl displaying heavy chain and light chain fragments.

a.)

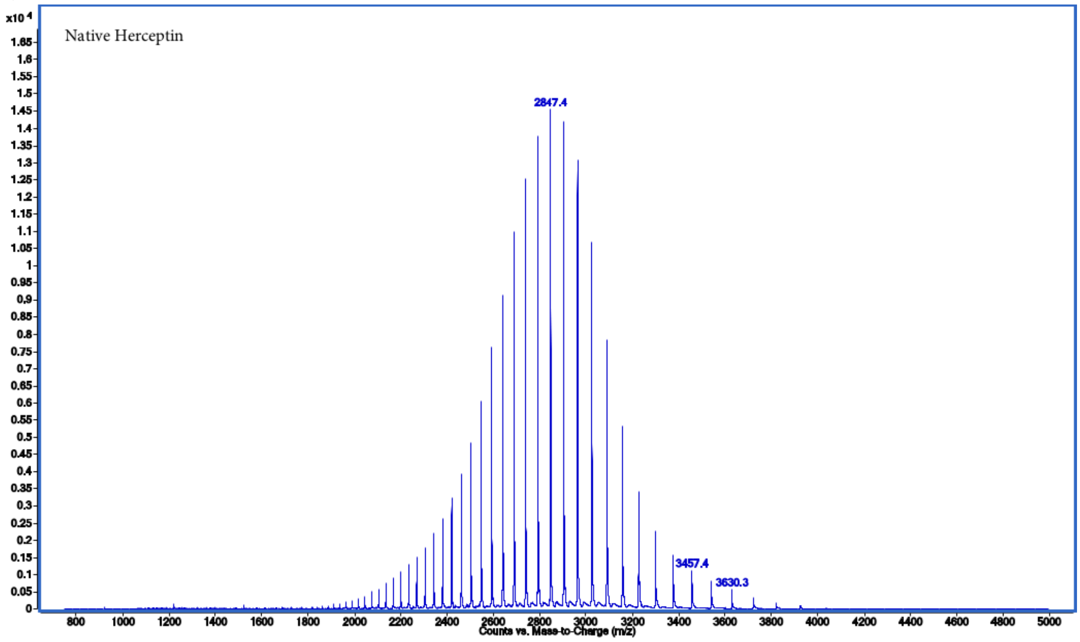

b.)

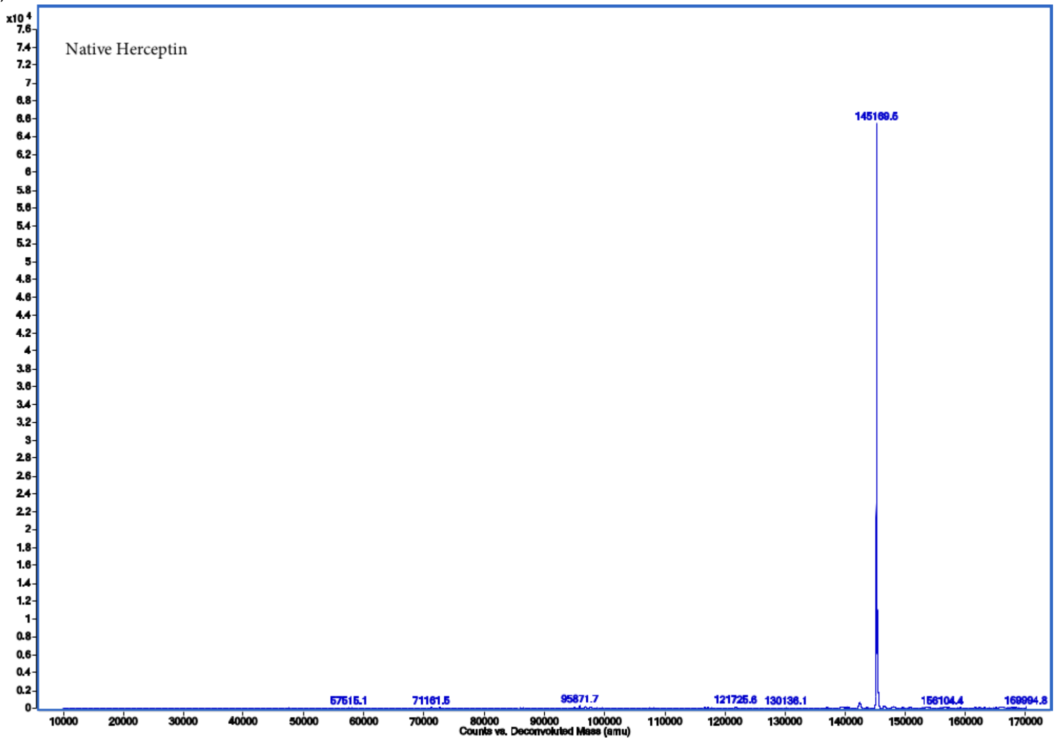

Figure **S14b**. (a) non-deconvoluted and (b) deconvoluted MS data for native Herceptin™ (deglycosylated).

a.)

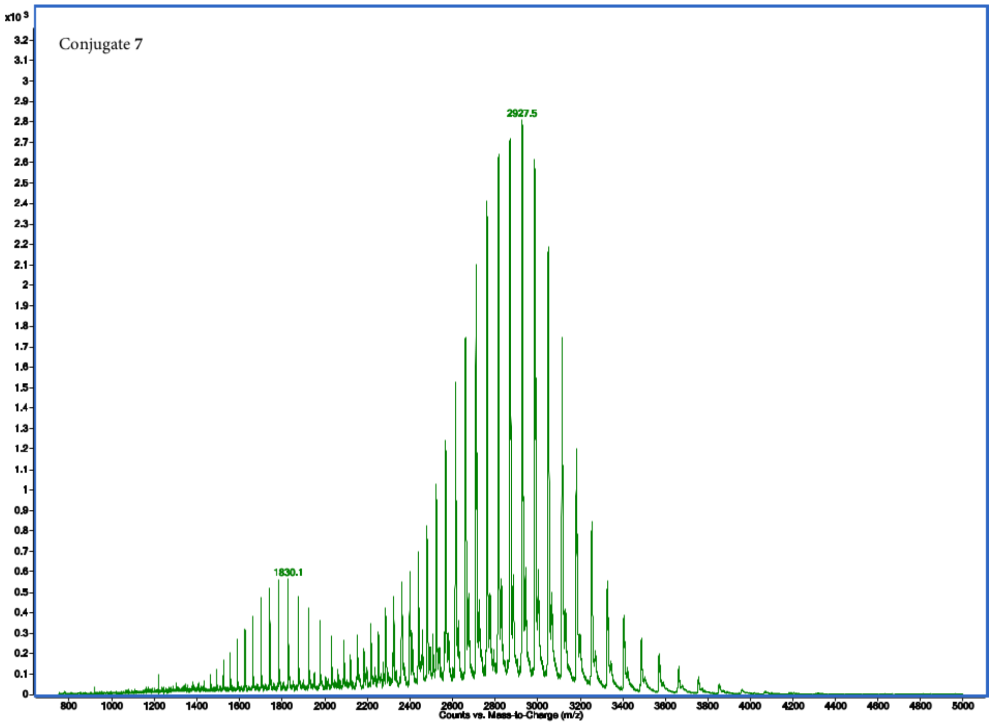

b.)

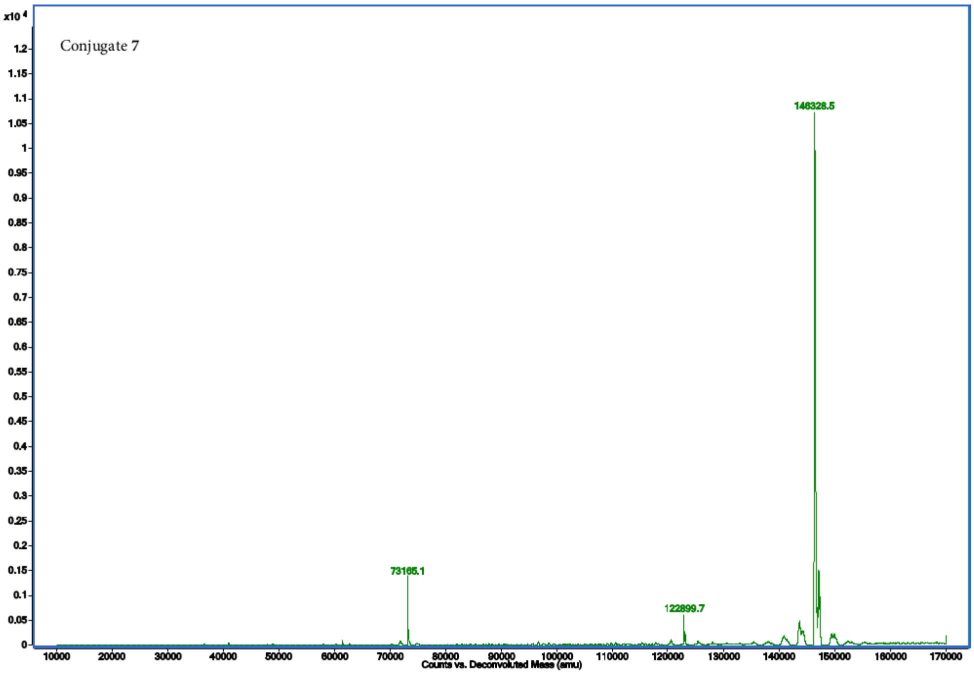

Figure **S14c**. (a) non-deconvoluted and (b) deconvoluted MS data for conjugate **7** (deglycosylated).

.

Reaction of conjugate 7 with AlexaFluor 488®-azide for the formation of conjugate 8

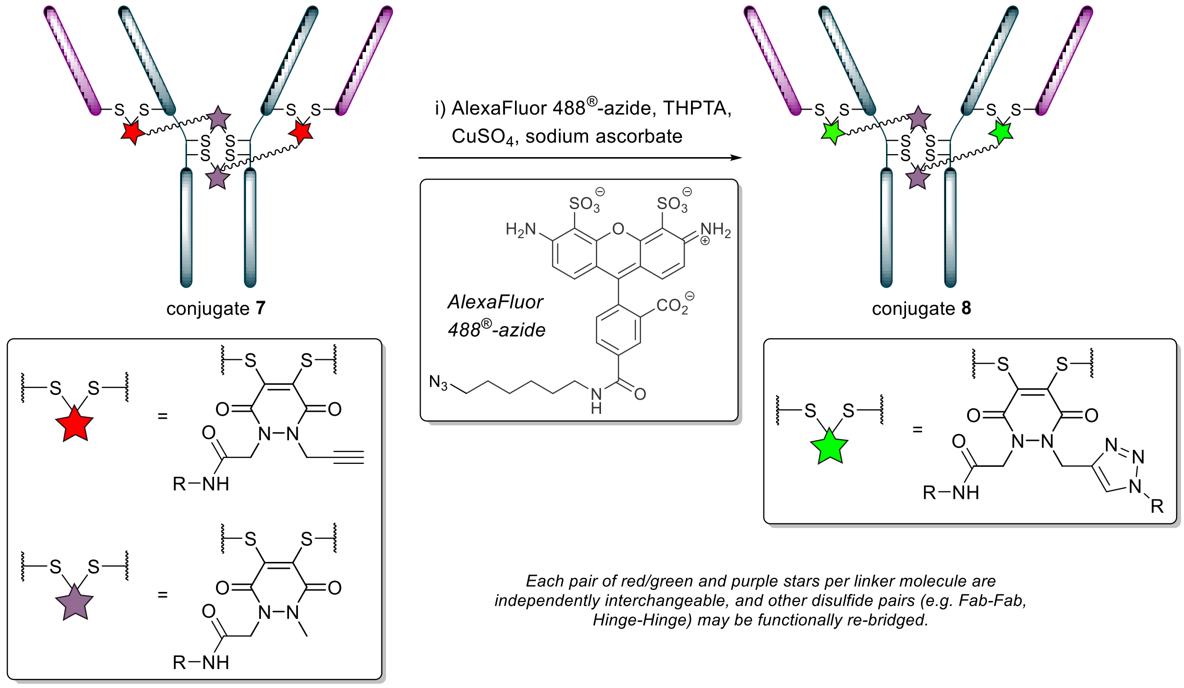

AlexaFluor 488®-azide (5.0 µL, 10.0 mM in DMSO, 20 eq.), $CuSO_4$ (1.0 µL, 20.0 mM in water, 8 eq.), tris(3-hydroxypropyltriazolylmethyl)amine (THPTA) (1.0 µL, 100 mM in water, 40 eq.) and sodium ascorbate (5.0 µL, 0.10 M in water) were added in series to conjugate **7** (50 µL, 7.5 mg/mL, 50 µM) in BBS (25 mM sodium borate, 25 mM NaCl, pH 8.0). The reaction mixture was stored at 21 °C for 1.5 h. The excess reagents were then removed by repeated diafiltration into fresh PBS with 2 mM EDTA (to remove residual copper ions) using VivaSpin sample concentrators (GE Healthcare, 10,000 MWCO). Following this, the buffer salts were removed by repeated diafiltration into deionised water using VivaSpin sample concentrators (GE Healthcare, 10,000 MWCO). The samples were analysed by SDS-PAGE gel and UV-Vis spectroscopy, which was used to determine a FAR of 2.0. This reaction was repeated on 7 separate occasions with remarkable reproducibility; FARs in the range of 1.9-2.1 were observed.

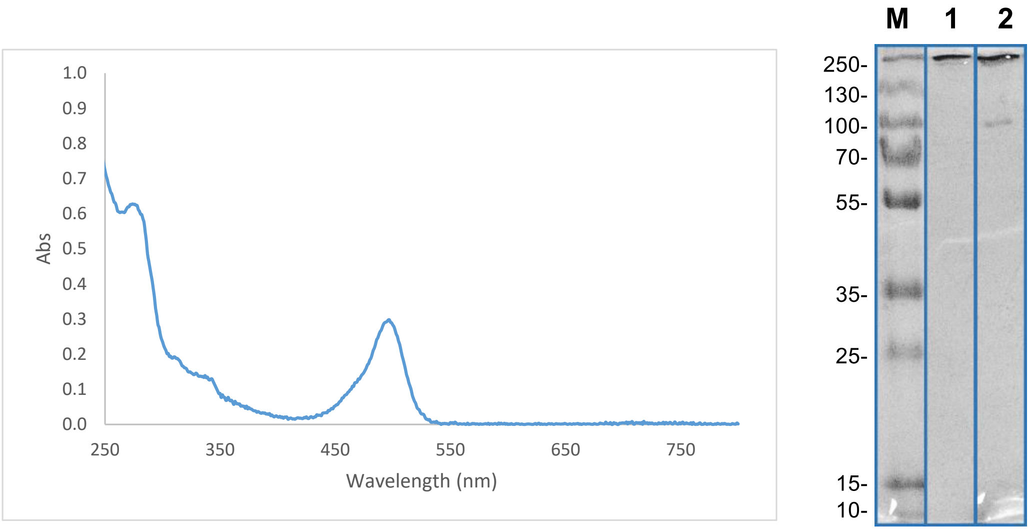

Figure **S15**. (Left) UV-Vis data for conjugate **8**. (Right) SDS-PAGE gel; M.) Molecular weight marker; 1) Unmodified Herceptin™; 2) Conjugate **8**.

Reaction of conjugate 7 with Doxorubicin-azide[2] for the formation of 'Her-Dox-DAR 2' conjugate

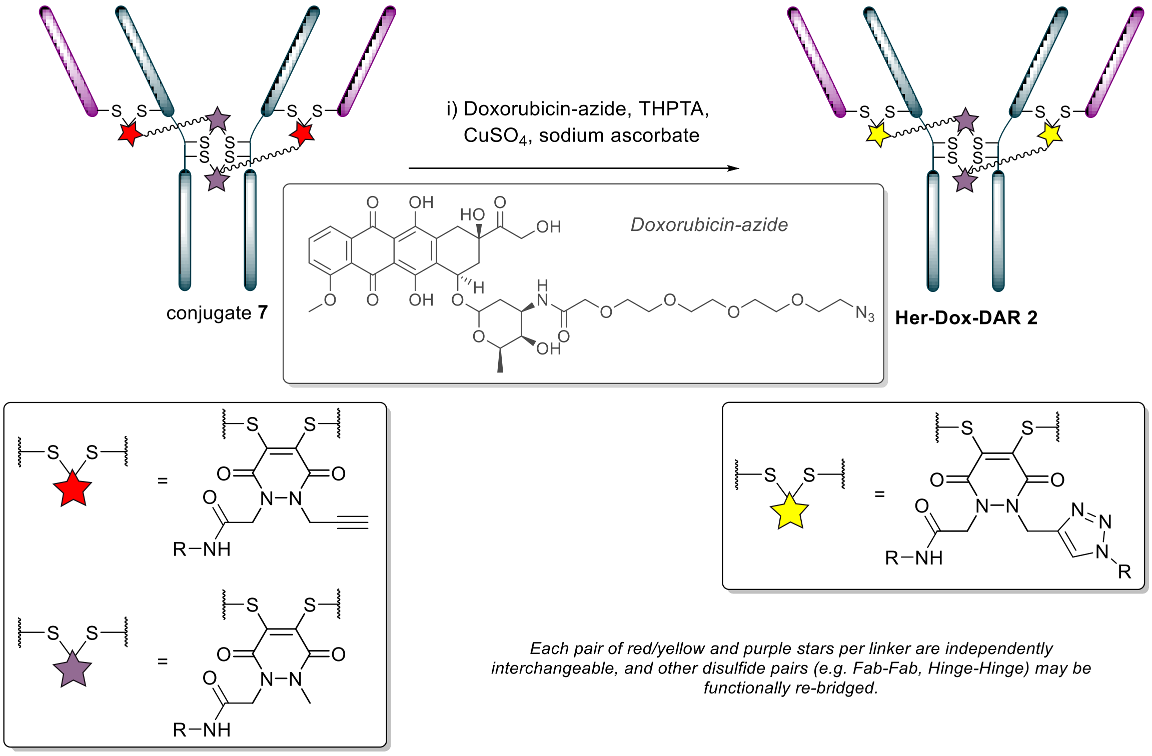

Doxorubicin-azide[2] (5.0 µL, 10.0 mM in DMSO, 20 eq.), $CuSO_4$ (1.0 µL, 20.0 mM in water, 8 eq.), tris(3-hydroxypropyltriazolylmethyl)amine (THPTA) (1.0 µL, 100 mM in water, 40 eq.) and sodium ascorbate (5.0 µL, 0.10 M in water) were added in series to conjugate **7** (50 µL, 7.5 mg/mL, 50 µM) in BBS (25 mM sodium borate, 25 mM NaCl, pH 8.0). The reaction mixture was stored at 21 °C for 1.5 h. The excess reagents were then removed by repeated diafiltration into fresh PBS with 2 mM EDTA (to remove residual copper ions) using VivaSpin sample concentrators (GE Healthcare, 10,000 MWCO). Following this, the buffer salts were removed by repeated diafiltration into deionised water using VivaSpin sample concentrators (GE Healthcare, 10,000 MWCO). The samples were analysed by SDS-PAGE gel and UV-Vis spectroscopy was used to determine a DAR of 2.0.

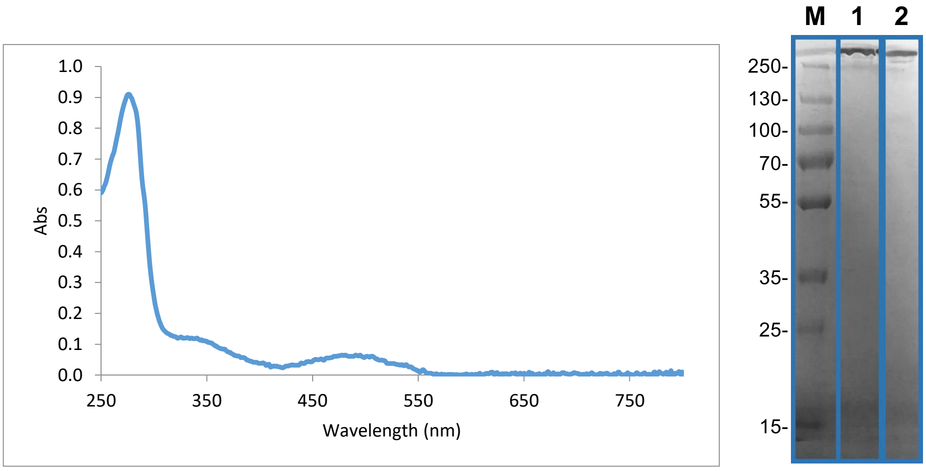

Figure **S16**. (Left) UV-Vis data for **Her-Dox-DAR 2**. (Right) SDS-PAGE gel; M.) Molecular weight marker; 1) Unmodified Herceptin™; 2) **Her-Dox-DAR 2**.

In situ reduction of Herceptin™ mAb and reaction with bis-diBrPD 9 at 4 °C (16 eq.) for the formation of 'Her-tet-Alk' conjugate

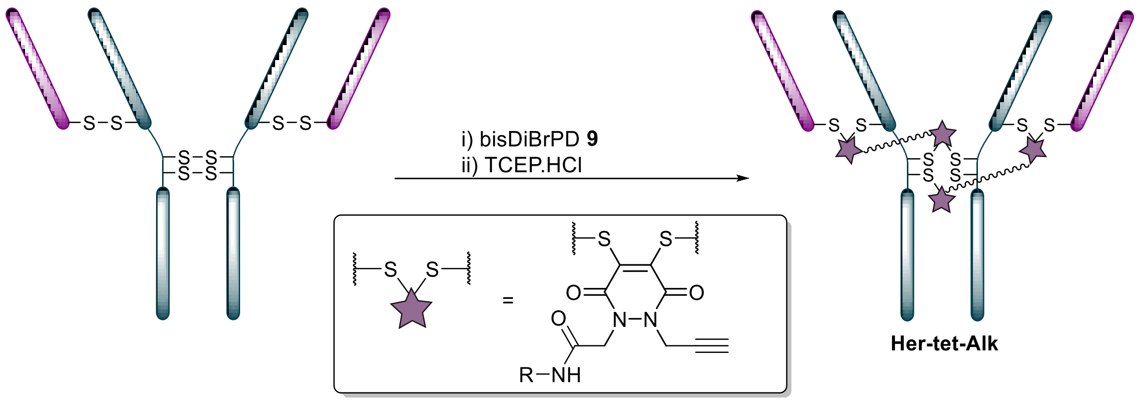

TCEP.HCl (10 µL, 20 mM in deionised water, 80 eq.) was added to a solution of Herceptin™ (50 µL, 7.5 mg/mL, 50 µM) in BBS (25 mM sodium borate, 25 mM NaCl, 0.5 mM EDTA, pH 8.0) which had been pre-treated with bis-diBrPD **9** (2.0 µL, 20 mM in DMSO, 16 eq.) and stored at 4 °C for 1 h previously. The reaction mixture was then stored at 4 °C for 15 h. Excess reagents were removed by repeated diafiltration into deionised water using VivaSpin sample concentrators (GE Healthcare, 10,000 MWCO). The samples were analysed by SDS-PAGE gel and UV-Vis spectroscopy was used to determine a PDAR of 3.9.

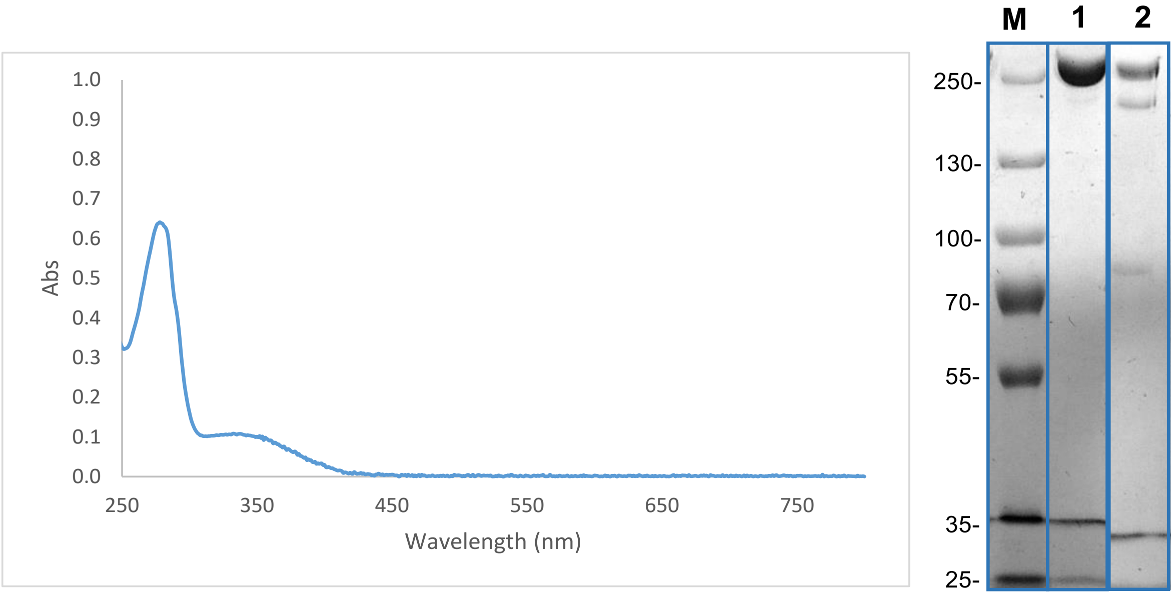

Figure **S17**. (Left) UV-Vis data for **Her-tet-Alk**. (Right) SDS-PAGE gel; M) Molecular weight marker; 1) Untreated Herceptin™; 2) **Her-tet-Alk**. Artefact in SDS-PAGE gel observed in both samples (_ca._ 35 kDa).

Reaction of Her-tet-Alk with AlexaFluor 488®-azide for the formation of conjugate 10

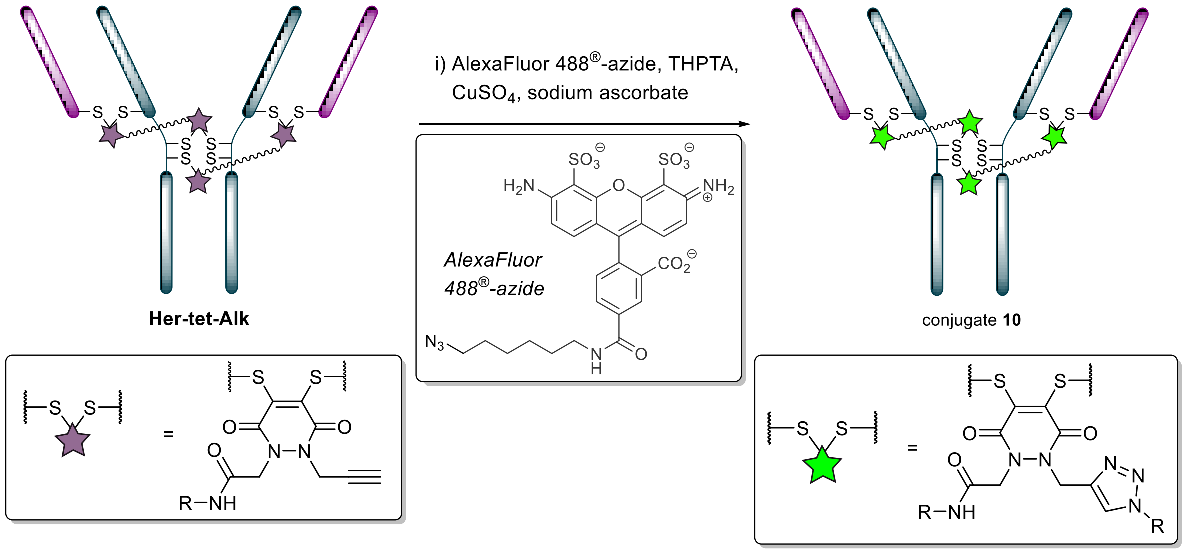

AlexaFluor 488®-azide (10.0 µL, 10.0 mM in DMSO, 40 eq.), $CuSO_4$ (1.0 µL, 20.0 mM in water, 8 eq.), tris(3-hydroxypropyltriazolylmethyl)amine (THPTA) (1.0 µL, 100 mM in water, 40 eq.) and sodium ascorbate (5.0 µL, 0.10 M in water) were added in series to **Her-tet-Alk** (50 µL, 7.5 mg/mL, 50 µM) in BBS (25 mM sodium borate, 25 mM NaCl, pH 8.0). The reaction mixture was stored at 21 °C for 1.5 h. The excess reagents were then removed by repeated diafiltration into fresh PBS with 2 mM EDTA (to remove residual copper ions) using VivaSpin sample concentrators (GE Healthcare, 10,000 MWCO). Following this, the buffer salts were removed by repeated diafiltration into deionised water using VivaSpin sample concentrators (GE Healthcare, 10,000 MWCO). The samples were analysed by SDS-PAGE gel and UV-Vis spectroscopy was used to determine a FAR of 3.9.

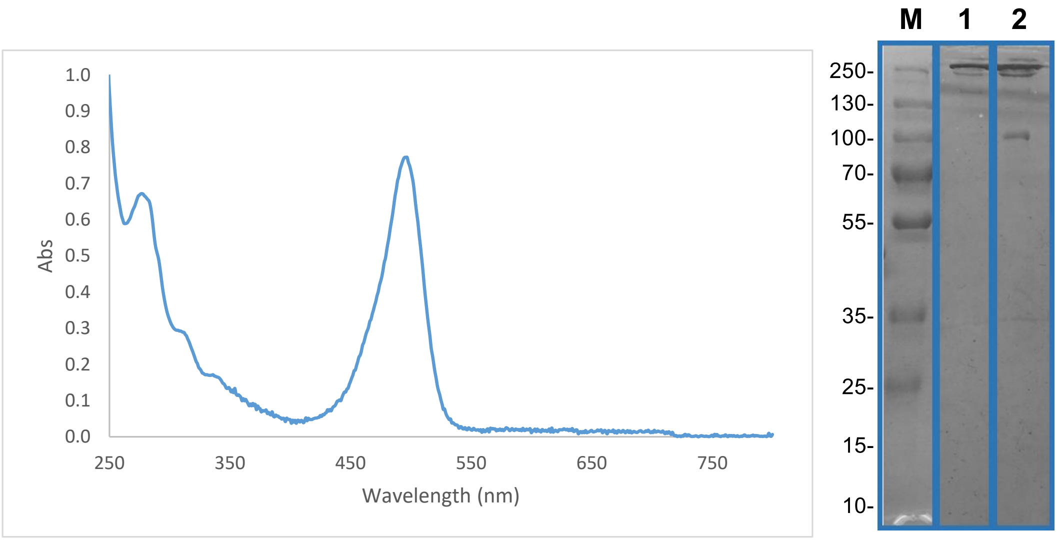

Figure **S18**. (Left) UV-Vis data for conjugate **10** modified with AlexaFluor 488®-azide. (Right) SDS-PAGE gel; M) Molecular weight marker; 1)Unmodified Herceptin™; 2) Conjugate **10**.

In situ reduction of Herceptin™ mAb and reaction with DiEt-PD at 4 °C (16 eq.) for the formation of 'Her-DiEt'

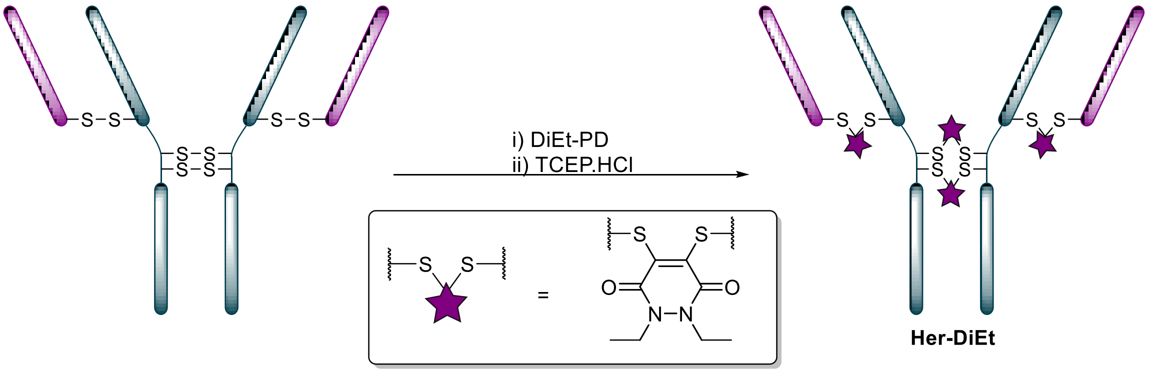

TCEP.HCl (2.3 µL, 20 mM in deionised water, 25 eq.) was added to Herceptin™ (30 µL, 4.4 mg/mL, 30 µM) in BBS (25 mM sodium borate, 25 mM NaCl, 0.5 mM EDTA, pH 8.0) which had been pretreated and stored at 4 °C for 1 h previously with DiEt-PD (3.6 µL, 4 mM in DMF, 16 eq.). The reaction mixture was then stored at 4 °C for 15 h. Excess reagents were removed by repeated diafiltration into deionised water using VivaSpin sample concentrators (GE Healthcare, 10,000 MWCO). The samples were analysed by SDS-PAGE gel and UV-Vis spectroscopy was used to determine a PDAR of 4.0.

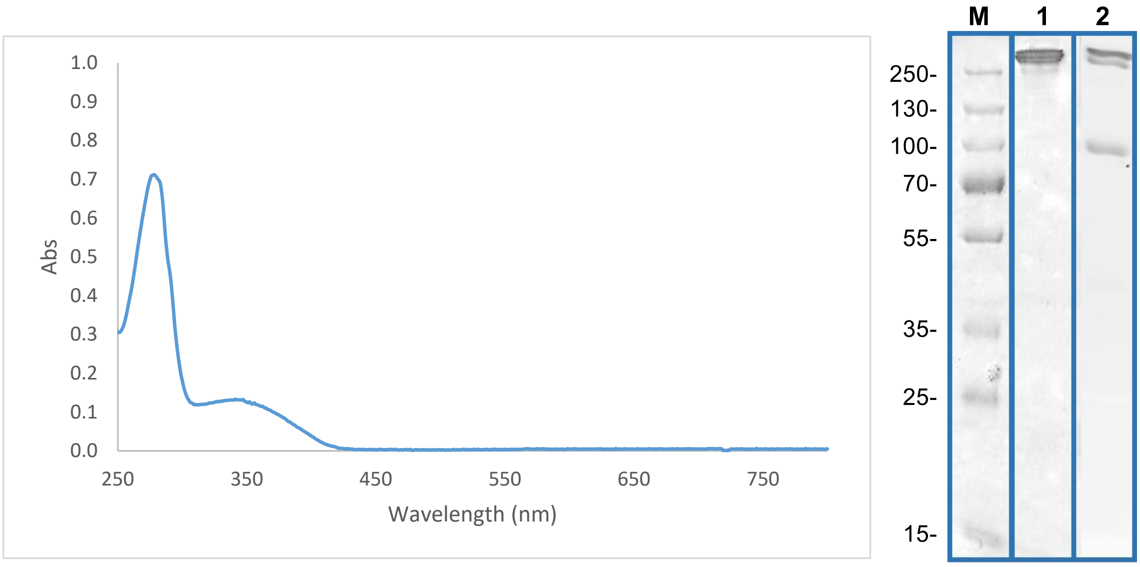

Figure **S19**. (Left) UV-Vis data for **Her-DiEt**. (Right) SDS-PAGE gel; M) Molecular weight marker; 1) Unmodified Herceptin™; 2) **Her-DiEt**.

Reduction of Herceptin™ mAb control for elucidation of accessible disulfides

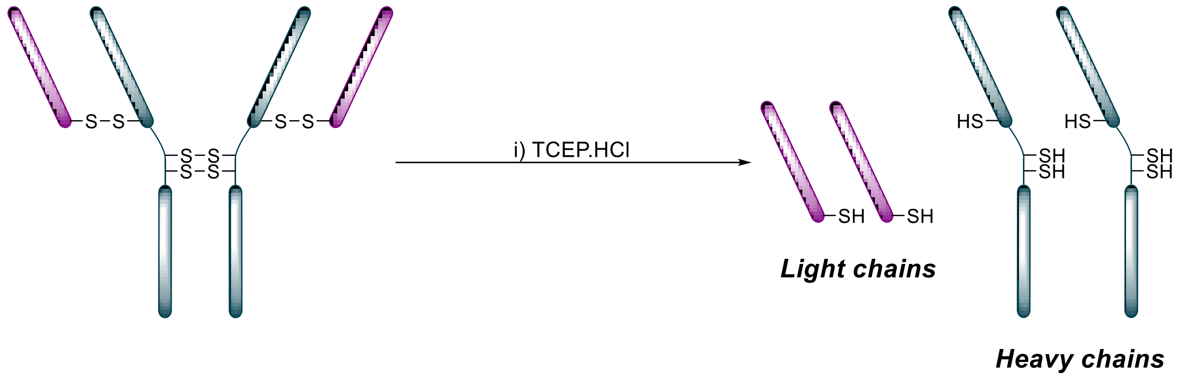

TCEP.HCl (10 µL, 20 mM in deionised water, 80 eq.) was added to Herceptin™ (50 µL, 7.5 mg/mL, 50 µM) in BBS (25 mM sodium borate, 25 mM NaCl, 0.5 mM EDTA, pH 8.0). The reaction mixture was then incubated at 37 °C for 1 h. The sample was analysed by SDS-PAGE gel revealing light chain and heavy chain fragments.

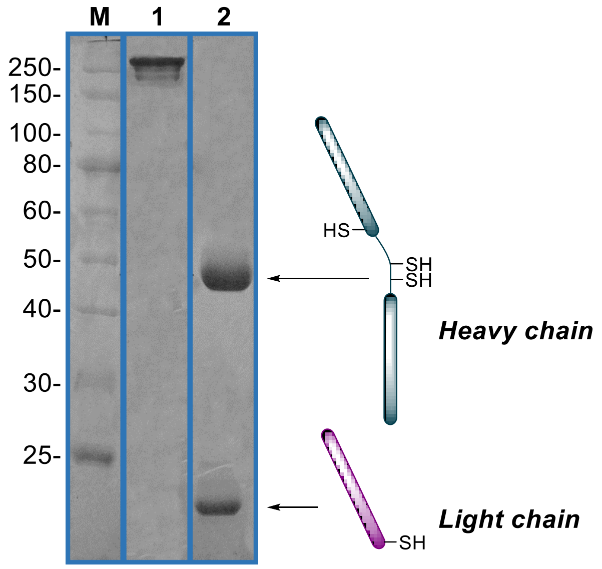

Figure **S20**. SDS-PAGE gel; M) Molecular weight marker; 1) Unmodified Herceptin™; 2) Herceptin™ reduced with TCEP.HCl displaying heavy chain and light chain fragments.

Activity of Conjugates by enzyme-linked immunosorbent assay (ELISA)

A 96-well Maxisorp plate was coated overnight at 4 °C with HER2 (0.25 μg·mL^{-1} in 50 mM sodium carbonate buffer pH = 9.6, 100 μl). As a negative control one row was coated with only buffer. The solutions were removed and each well washed (2 × PBS with 0.05% Tween). The wells were subsequently coated with a 1% BSA solution in PBS for one hour at room temperature. After this the wells were emptied and washed (4 × PBS with 0.05% Tween). Solutions of Native Herceptin™, Herceptin™ DiEt, conjugate **8,** and conjugate **10** in PBS pH = 7.4 were prepared in the following dilutions: 60.0 nM, 15.0 nM, 3.75 nM, 0.938 nM, 0.234 nM and 0.00586 nM. The dilutions were placed into the wells, each in triplicate, and incubated for two hours at room temperature. As negative controls sodium carbonate buffer only and the antibodies at 60 nM in the absence of HER2 were also subjected to the same protocol. The solutions were removed and the wells washed (4 × PBS with 0.05% Tween). Detection antibody (100 μL of anti-human IgG, Fab-specific-HRP solution, 4 μL of a 1:5000 solution diluted further in 20 ml of PBS) was added and left for one hour at room temperature. The solutions were removed and the wells washed (4 × PBS with 0.05% Tween). Finally, a TMB solution (1 × TMB Solution, eBioscience, 100 μL) was added to each well. After five minutes the reaction was stopped through addition of 0.2 M sulfuric acid (50 μL). Absorbance was measured at 450 nm and corrected by subtracting the average of negative controls. Protocol was adapted from a literature procedure.[2]

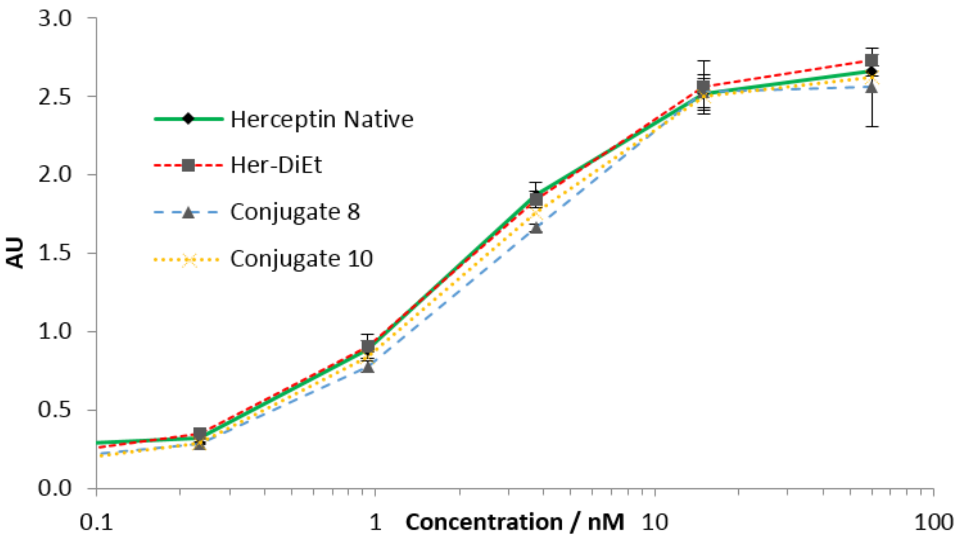

Figure **S21**. Binding activity of conjugates **8, 10** and **Her-DiEt** against unmodified Herceptin™**.**

Reaction of bis-DiBrPD 1 with AlexaFluor 488®-azide for the formation of bis-DiBrPD-AF488 and subsequent reaction with Herceptin™ for the formation of conjugate 8A

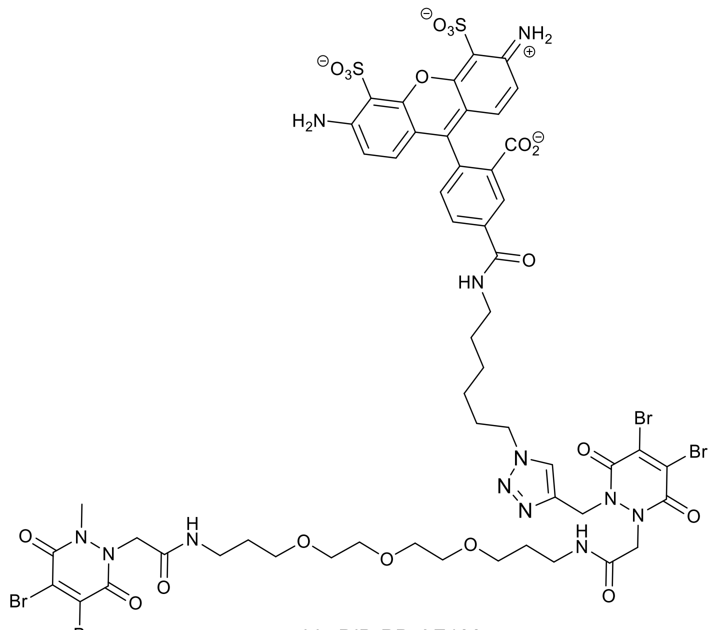

bis-DiBrPD-AF488

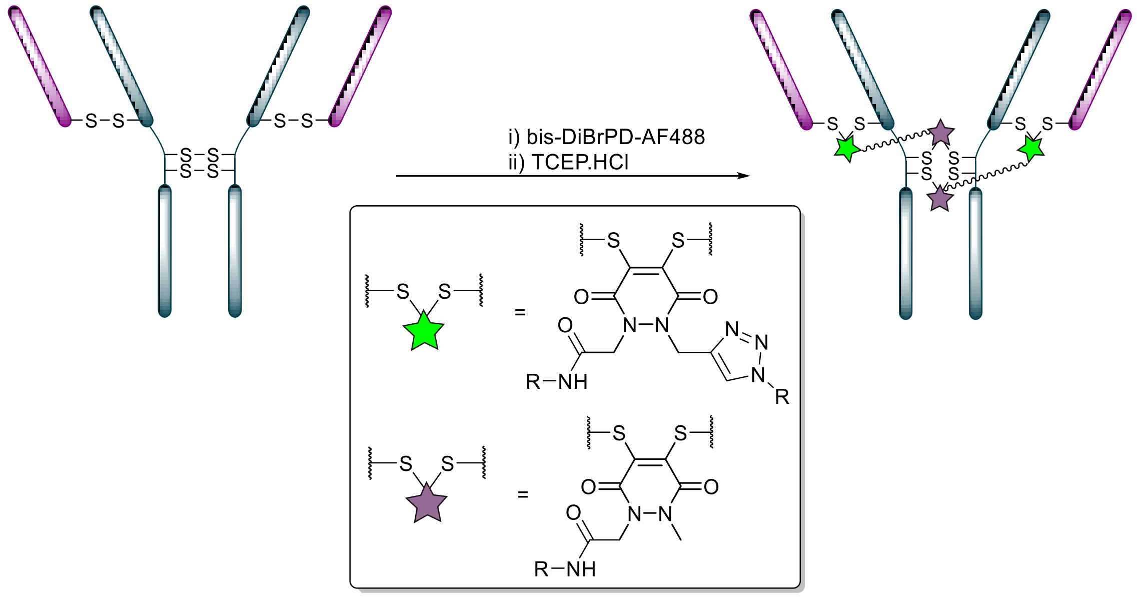

Each pair of green and purple stars per linker molecule are independently interchangeable, and other disulfide pairs (e.g. Fab-Fab, Hinge-Hinge) may be functionally re-bridged.

AlexaFluor 488®-azide (7.5 µL, 10.0 mM in DMSO, 5 eq.), $CuSO_4$ (3.0 µL, 20.0 mM in water, 4 eq.), tris(3-hydroxypropyltriazolylmethyl)amine (THPTA) (3.0 µL, 100 mM in water, 20 eq.) and sodium ascorbate (5.0 µL, 0.10 M in water) were added in series to bis-DiBrPD **1** (10 µL, 1.5 mM) in DMSO. The reaction mixture was stored at 21 °C for 1.5 h. After this time the reaction was quenched with EDTA (7.2 µL, 0.5 M in water). Following this an aliquot of the reaction mixture containing the

newly formed bis-DiBrPD-AF488 (19.0 µL, 526 µM) was added to a solution of Herceptin™ (50 µL, 1.88 mg/mL, 12.5 µM) in BBS (25 mM sodium borate, 25 mM NaCl, 0.5 mM EDTA, pH 8.0) and stored at 4 °C for 1 h. After this time TCEP.HCl (5 µL, 10.0 mM in deionised water, 80 eq.) was added and the reaction mixture was then stored at 4 °C for a further 15 h. Excess reagents were removed by repeated diafiltration into deionised water using VivaSpin sample concentrators (GE Healthcare, 10,000 MWCO). The samples were analysed by SDS-PAGE gel and UV-Vis spectroscopy, which was used to determine a FAR of 1.8.

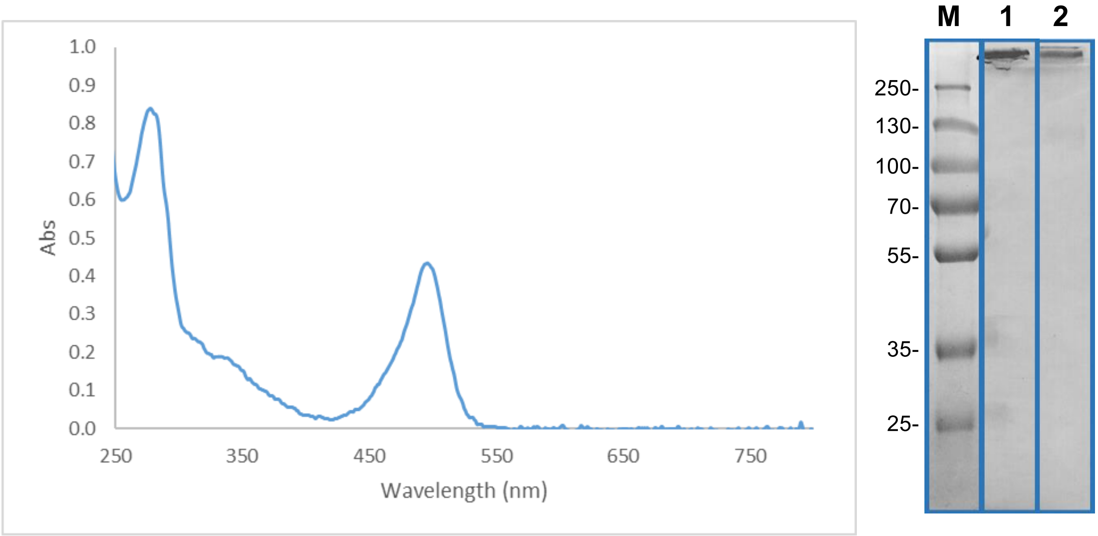

Figure **S22**. (Left) UV-Vis data for conjugate **8A**. (Right) SDS-PAGE gel; M) Molecular weight marker; 1) Unmodified Herceptin™; 2) Conjugate **8A**.

References

1. A. Maruani, H. Savoie, F. Bryden, *et al.*, *Chem. Commun.*, 2015, **51**, 15304-15307.
2. A. Maruani, M. E. B. Smith, E. Miranda, *et al.*, *Nat. Commun.*, 2015, **6**.
3. J. C. Slootweg, S. van der Wal, H. C. Quarles van Ufford, *et al.*, *Bioconjugate Chem.*, 2013, **24**, 2058-2066.
4. M. T. W. Lee, A. Maruani, J. Baker, *et al.*, *Chem. Sci.*, 2016, **7**, 799-802.
